# Recent perspectives on the synergy of mesenchymal stem cells with micro/nano strategies in peripheral nerve regeneration-a review

**DOI:** 10.3389/fbioe.2024.1401512

**Published:** 2024-07-10

**Authors:** Majid Sharifi, Mohammad Kamalabadi-Farahani, Majid Salehi, Somayeh Ebrahimi-Brough, Morteza Alizadeh

**Affiliations:** ^1^ Student Research Committee, School of Medicine, Shahroud University of Medical Sciences, Shahroud, Iran; ^2^ Tissue Engineering and Stem Cells Research Center, Shahroud University of Medical Sciences, Shahroud, Iran; ^3^ Department of Tissue Engineering, School of Medicine, Shahroud University of Medical Sciences, Shahroud, Iran; ^4^ Health Technology Incubator Center, Shahroud University of Medical Sciences, Shahroud, Iran; ^5^ Department of Tissue Engineering and Applied Cell Sciences, School of Advanced Technologies in Medicine, Tehran University of Medical Sciences, Tehran, Iran; ^6^ Department of Tissue Engineering and Biomaterials, School of Advanced Medical Sciences and Technologies, Hamadan University of Medical Sciences, Hamadan, Iran

**Keywords:** MSCs, peripheral nerve, Neuroprotection, neuroregeneration, nanostructures

## Abstract

Despite the intrinsic repair of peripheral nerve injury (PNI), it is important to carefully monitor the process of peripheral nerve repair, as peripheral nerve regeneration is slow and incomplete in large traumatic lesions. Hence, mesenchymal stem cells (MSCs) with protective and regenerative functions are utilized in synergy with innovative micro/nano technologies to enhance the regeneration process of peripheral nerves. Nonetheless, as MSCs are assessed using standard regenerative criteria including sensory–motor indices, structural features, and morphology, it is challenging to differentiate between the protective and regenerative impacts of MSCs on neural tissue. This study aims to analyze the process of nerve regeneration, particularly the performance of MSCs with and without synergistic approaches. It also focuses on the paracrine secretions of MSCs and their conversion into neurons with functional properties that influence nerve regeneration after PNI. Furthermore, the study explores new ideas for nerve regeneration after PNI by considering the synergistic effect of MSCs and therapeutic compounds, neuronal cell derivatives, biological or polymeric conduits, organic/inorganic nanoparticles, and electrical stimulation. Finally, the study highlights the main obstacles to developing synergy in nerve regeneration after PNI and aims to open new windows based on recent advances in neural tissue regeneration.

## 1 Introduction

**FIGURE 1:**
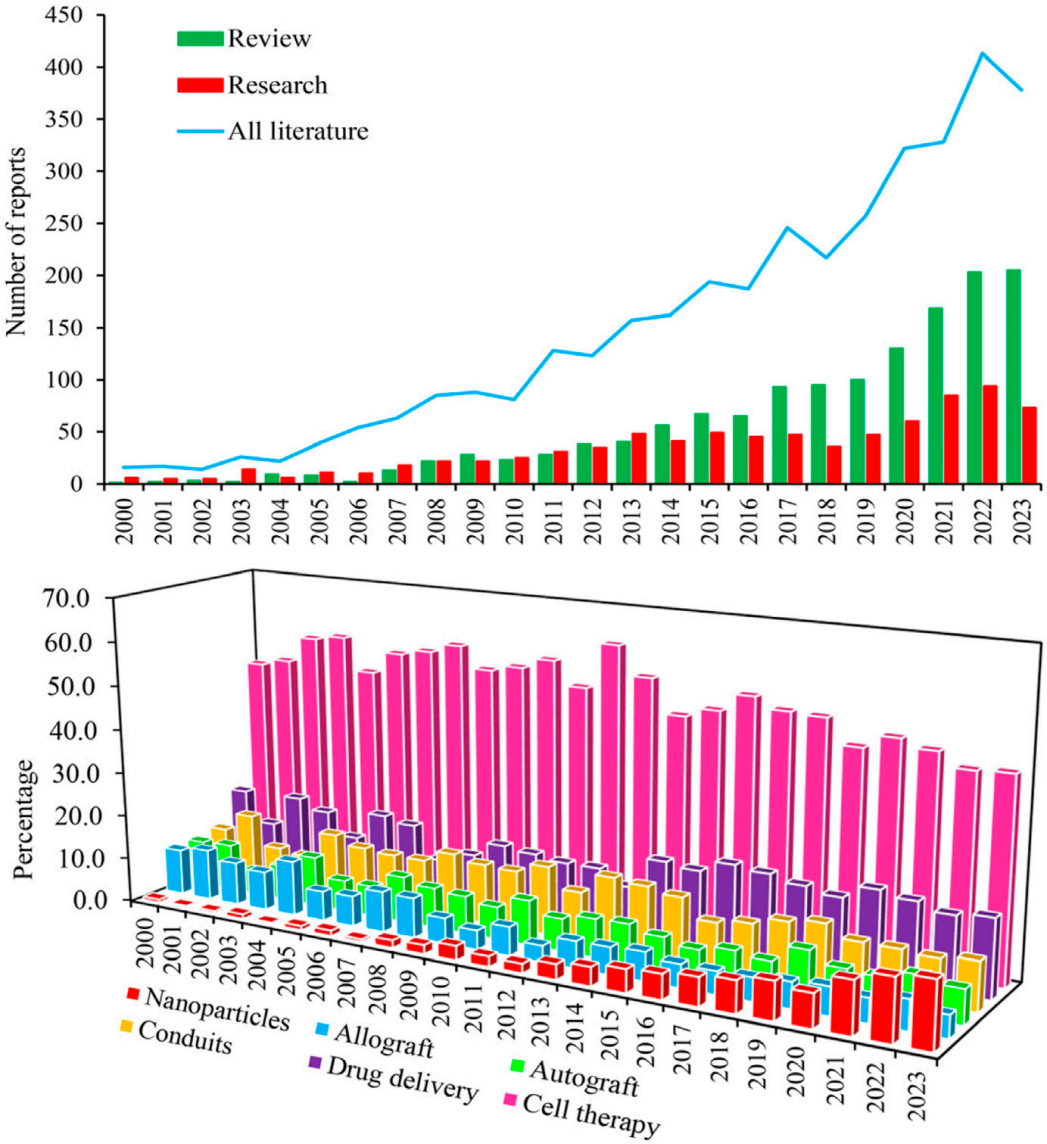
Chronological increase of scientific interest in the use of MSCs with and without neural progenitor cells in the treatment of PNI, as indicated by research and review article counts in Scopus, PubMed, and Web of Science (Upper panel). Interest percentage in the utilization of MSCs for repairing PNI based on various therapeutic strategies between 2000 and 2023 (Lower panel).

Despite the long history of peripheral nerve (PN) regeneration through therapeutic interventions since the early19^th^ century (Todd, 1823), sensory-motor disorders resulting from peripheral nerve injury (PNI) remain a major challenge in human society. PN have a greater capacity for repair than the central nervous system. Nevertheless, self-repair of PN may result in secondary complications including dysfunction, pain, decreased surgical effectiveness, scarring, adhesions, and neuromas depending on the site, type, and seriousness of the injury (Scheib and Höke, 2013; Panagopoulos et al., 2017; Ortiz et al., 2022). Thus, scientists are exploring different methods like cell therapy, nanomedicine, and drug delivery to reduce complications and enhance the PNs self-renewal rate (Figure 1; Table 1), particularly when the nerve is severed over 5 mm. However, these strategies are challenging to apply to neuron regeneration due to the limitations outlined in Table 1.

**TABLE 1:** The most common strategies used in peripheral nerve regeneration after PNI (Scheib and Höke, 2013; Chan et al., 2014; Wang et al., 2015; Kornfeld et al., 2019; Vijayavenkataraman, 2020; Sharifi et al., 2022b; Supra et al., 2023).

Therapeutic strategy		Benefits	Drawbacks
Chemotherapy		Different treatment routes, high drug diversity, high synergy with other therapeutic approaches, simultaneous use of different drug compounds, simple therapeutic management, relatively low cost	Low targeting, high side effects with long-term therapy, toxicity from overdose, low drug stability, low regenerative efficiency and weak neurologic function, lack of control over the regeneration process, low neurogenesis, long-term treatment
Cell therapy		Improves the regeneration environment with programmable secretions, ability to control immunogenesis, ability to synchronize therapeutic perspectives, optimal therapeutic efficiency compared with chemotherapy, low neuropathy, low invasiveness	Tumorigenesis and teratogenicity in pluripotent stem cells, high costs, pre-treatment and transfer, ethical challenges in some cells such as ESCs, limited cell variety, lack of universal cells, dedifferentiation and unfavorable differentiation, limited commercialization, side effects with migrating, conflicting therapeutic responses
Grafts	Autograft	Accelerates regeneration, no immunogenicity, providing neurotrophic factors, easy access, no graft rejection, easy suturing, rapid inflammation reduction	Resource limitations, multiple surgeries, donor site challenges such as trauma, infection and tissue inefficiency, prolonged treatment, neuroma formation, different tissue size
	Allograft	Reduces surgical time and improved recovery time, useful in large nerve damage, access higher than autograft	Acceleration of immunogenicity, virus, or bacteria transmission, neuro-structural changes during processing, relative decrease in neurological function, resource limitations, ethical concerns, need for suppressors
Conduits	Biological	Improves neural structure, strong cell bonding, reservoir of neurotrophic factors, biodegradable, nerve buds’ guidance, enhanced angiogenesis, low inflammation	Diverse neural responses, long manufacturing process, more limited access, in some cases immunological challenges
	Natural	Provides cell adhesion agents, biodegradable, significant control of fibrosis, control of cell migration, semi-porous, relatively cheap, improves angiogenesis in some cases	Low Young`s modulus, variable mechanical properties, asymmetric degradability, limited resources, high inflammatory reactivity, high decomposition at pH < 7, heterogenous structure
	Synthetic	Controllable and reproducible physicochemical structures, high mechanical features, high porosity and permeability, cell migration enhancement, simple processing, low cost	Low cell adhesion, toxicity byproducts, low bioactivity, low biocompatibility, risk of nerve compression during repair, ischemia of adjacent tissues, reduced angiogenesis, poor repeatability in production
Nanoparticles	Inorganic	Regulates cell migration, induces physicochemical signals, antibacterial, increases electrical conductivity, guiding the growth of neurites and axons, biocompatible	High toxicity due to agglomeration or release of active ions, long-term stability in damaged tissue, impurities in some alloys, immunogenicity, non-bioactivity
	Organic	High biocompatibility, high cellular attachment, bioactivity, good availability, low cost, inducing cell differentiation, biodegradable, limited immunogenicity	Impurities in some sources, limitation in the engineering of platforms with different shapes and dimensions, Rapid degradation in some materials, immunogenicity, ambiguity in inducing growth of nerves and axons
	Exosomes	Low immunogenicity, highly targeted, enhanced neurogenesis by inducing biological agents, easy maintenance, few side effects, biocompatibility	Lack of standard manufacturing protocol, low stability, difficult to isolate and purify, conflicting reaction in neuronal regeneration, low reproducibility due to different molecular profile

To address mentioned obstacles, researchers are now focusing on synergistic strategies that combine multiple techniques and interventions. For instance, neural tissue regeneration can be stimulated by the synergistic effects of mesenchymal stem cells (MSCs) or their secretomes with conduits to reduce inflammation and optimize the environment (Shalaby et al., 2017). Also, the use of nanoparticles (NPs) with MSCs to promote their proliferation and conversion into neurons is of great interest (Tseng and Hsu, 2014). These activities have shown positive impacts on PN regeneration after injury by regulating growth factors, cell exchange, and migration control. However, accessing neural progenitor cells during PN regeneration remains a specific priority (Sullivan et al., 2016). Despite the great effects of MSCs in reducing inflammation, promoting the proliferation of niche cells, and increasing the neural progenitor cell function, their use remains challenging (Zhang R.-C. et al., 2021; Lavorato et al., 2021). One challenge is the lack of promising neuronal function and an insufficient number of neurites after differentiation (Gu et al., 2017). Nevertheless, researchers are actively working to address these challenges and improve the efficacy of MSC-based PN regeneration. The impressive synergistic effects of MSCs on neuronal regeneration have led to increasing application of MSCs, with or without neural progenitor cells. Since the discovery of bone marrow MSCs (BM-MSCs) in 1970 (Charbord, 2010), MSCs have attracted considerable attention in regenerative medicine due to several potential features including selective differentiation, reduced inflammation, abundant resources, and simple and easy extraction (Sensharma et al., 2017). Despite these benefits, the protective effect of MSCs on the neural microenvironment and their differentiation into neural cells and structures remains unknown. Therefore, this review aims to provide a perspective on PN regeneration based on the synergistic effect of MSCs and micro- and nano-structural strategies. Also, this review attempts to highlight the challenges, benefits, and limitations associated with synergistic strategies in MSC-based PN regeneration.

## 2 Mesenchymal stem cells

**FIGURE 2:**
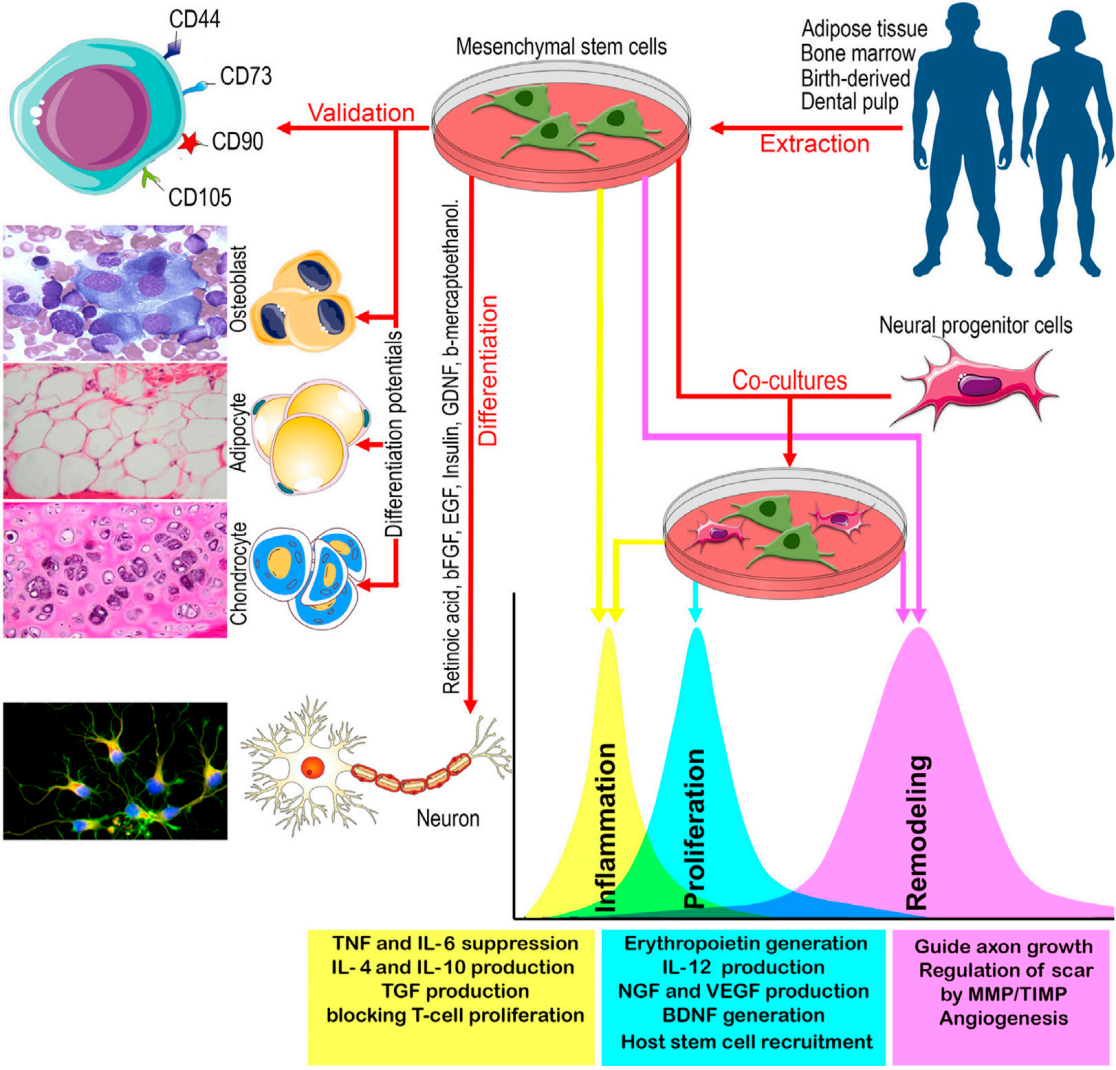
A schematic view of the main sources of MSCs, their validation, differentiation, and co-culture with neural progenitor cells, and the effects of MSCs with and without neural progenitor cells on inflammation, proliferation, and remodeling of PN. BDNF: Brain derived neurotrophic factor, bFGF: Basic fibroblast growth factor, EGF: Epidermal growth factor, GDNF: Glial-derived neurotrophic factor, IL: Interleukin, MMP: Matrix metalloproteinase, NGF: Nerve growth factor, TGF: Tumor growth factor, TIMP: tissue inhibitors of metalloproteinase, TNF: Tumor necrosis factor, VEGF: Vascular Endothelial Cell Growth Factor.

In PNI repairs, autologous, allogeneic, or xenogeneic stem cells are typically isolated, cultured, and then transferred to the injured area. In this context, MSCs are highly valued for their abundant available resources, lack of ethical issues, low immunogenicity, pronounced anti-inflammatory function, and simultaneous multimodal functions (Berebichez-Fridman and Montero-Olvera, 2018) (Table 2). Mesoderm-derived and multipotent MSCs (Jeon et al., 2015) employ three strategies for PN regeneration: (1) secretion of biological factors, (2) housekeeping approaches, and (3) multimodal differentiation potential (Figure 2). However, contrary to the *in vitro* results, *in vivo* outcomes indicate that MSCs, instead of differentiating into damaged tissue cells, contribute to the formation and training of neural progenitor cells. Thus, deciphering the function of MSCs with and without neural progenitor cells in PN regeneration requires the identification of MSC-specific markers and their differentiated cells (Table 2).

**TABLE 2:** Sources, extraction, differences, and the characteristics of MSCs.

Cells	Source	Extraction	Drawbacks	Markers	Differentiation capabilities	Ref.
Bone marrow-MSCs	Tubular, iliac crest, femur, tibia	Washing the bone marrow, separating the cells by centrifugation, and removing non-adherent cells in the culture medium	Extracting MSCs from this source is painful and the risk of transmission of infection is serious. The capacity and volume of the cell depends on the age of the donor	CD29^+^, CD44^+^, CD73^+^, CD90^+^, CD105^+^, Sca-1^+^, CD14^−^, CD34^−^, CD45^−^, CD19^−^, CD11b^−^, CD31^−^, CD86^−^	Adipocytes, Astrocytes, Cardiomyocytes, Chondrocytes, Hepatocytes, Mesangial cells, Muscle cells, Neurons, Osteoblasts, Stromal cells	Charbord (2010), Kassis et al. (2011), Sharifi et al. (2022c)
Adipose-MSCs	Subcutaneous adipose, buttocks, and abdominal zone	Digesting the fragmented tissue with type I collagenase and centrifuging them, then culturing the cells to remove non-adherent cells	Despite the easy access and the high number of cells that can be extracted, it has a low differentiation potential to bone, liver, nerve, and heart tissues	CD29^+^, CD34^+^, CD44^+^, CD73^+^, CD90^+^, CD105^+^, CD146^+^, CD166^+^, MHC-I^+^, CD31^−^, CD45^−^, CD117^−^, HLA-DR^−^	Adipocytes, Chondrocytes, Osteocytes, Muscle cells	Minteer et al. (2013), Zack-Williams et al. (2015)
Birth derived-MSCs	Umbilical cord blood (UCB), placenta (P), Warton’s Jelly (WJ), amniotic fluid (AF)	1. Collection of umbilical cord blood by ficoll gradient, culture, and removal of non-adherent cells 2. Digestion of amniotic membranes or placenta by collagenase type 1 and collection of adherent cells from the culture medium	Although there are no ethical issues and noninvasive access, the differentiation potential is low. In addition, the reduction in the number of colonies and insufficient amount for clinical application is also significant	CD29^+^, CD44^+^, CD73^+^, CD90^+^, CD105^+^, CD166^+^, CD14^−^, CD31^−^, CD34^−^, CD45^−^, CD106^−^, HLA-DR^−^	UCB-MSCs: Adipocytes, Chondrocytes WJ-MSCs Chondrocytes Dopaminergic neurons P-MSCs Pancreatic cells AF-MSCs Neural stem cells Adipocytes Osteoblasts Chondrocytes Hepatocytes	Kim et al. (2014), Li et al. (2015), Lobov et al. (2024)
Skeletal-muscle-derived-MSCs	Skeletal muscle tissue	Enzymatic digestion of fragmented samples with type II collagenase and filtration with 40 or 100 μm filters and removal of non-adherent cells on the plastic surface	The cell harvesting approach is invasive and sometimes associated with the induction of infection	CD29^+^, CD44^+^, CD73^+^, CD90^+^, CD105^+^, CD14^−^, CD19^−^, CD34^−^, CD45^−^, HLA-DR^−^	Bone cells, Adipocytes, Chondrocytes, Muscle cells, Neural cells, Hepatocytes, Blood cells	Jackson et al. (2010), Musavi et al. (2018)
Skin-MSCs	Foreskin and skin biopsies	Dissection of cultured skin sample in DMEM to purify the cells attached to the bottom of the flask	Collecting samples by invasive methods, increasing the possibility of infection after specimen collection	CD44 ^+^, CD73 ^+^, CD90 ^+^, CD105 ^+^, CD166 ^+^, SSEA-4 ^+^, Vimentin ^+^, CD34 ^−^, CD45 ^−^, HLA-DR ^-^	Chondral cells, Bone cells, Adipocytes, Neural cells, Glial cells, Pancreatic cells, Smooth muscle cells	Park et al. (2012), Orciani and Di Primio (2013)
Dental pulp-MSCs	Wisdom teeth, ectopic or even decayed teeth or root canal surgery	Drain the pulp cavity with PBS and culture in DMEM-F12 to remove non-adherent cells	Despite the challenges in accessing ectomesenchymal and periodontal tissues, such as the limitation in the number of waste teeth or the invasiveness of the donation process, they are valuable due to their strong potential for differentiation into neuronal lineage	CD29^+^, CD44^+^, CD90^+^, CD105^+^, CD14^−^, CD34^−^, CD45^−^	Odontoblasts, Osteoblasts, Adipocytes, Chondrocytes, Neurogenic cells, Myogenic cells	Kawashima et al. (2017), Pisciotta et al. (2020), Sramkó et al. (2023)

In addition to specific markers, it is important to consider cell sources based on function and access. Aside from those in Table 2, neural crest-derived cells (NCCs) with MSC traits may enhance MSC conversion to neurons through dedifferentiation and transdifferentiation methods. To confirm this finding, it was shown that BM-MSCs have two developmental origins, one of which is neural crest, based on the NCC-specific codes P0-Cre/Floxed-EGFP and Wnt1-Cre/Floxed-EGFP (Morikawa et al., 2009). Those cells carrying neural crest stem cell genes clearly differentiated into neurons and glial cells. Subsequently, it was discovered that about 90% of gingival MSCs (G-MSCs) come from NCCs and 10% come from mesoderm. The NCC-derived G-MSCs have a strong capacity to differentiate into neurons and trigger apoptosis in activated T cells (Xu et al., 2013). In another study, Isern et al. (2014) demonstrated that Nestin^+^ cells, originating from resident NCCs in the bone marrow, sustain MSC activity and play a role in the generation of hematopoietic stem cells. They found that the MSCs that are responsible for hematopoietic stem cell formation have a shared lineage with peripheral sympathetic neurons and glial cells. However, NCC-derived MSCs were found to have distinct transcriptional and functional characteristics compared to mesodermal MSCs (Srinivasan et al., 2018). Although MSCs show phenotypic and functional diversity based on their origin, the genetic reasons and functional abilities behind these differences are not well comprehended.

## 3 Strategies for MSC-Based PN regeneration

MSCs show strong paracrine potential and their secretion can be responsible for nerve regeneration. Indeed, MSCs can stimulate the proliferation and differentiation of various cell types. Cell-to-cell contacts and paracrine signaling modulate the active molecule secretory capabilities of MSCs and stimulate the secretory activity of endogenous Schwann cells and the accumulation of macrophages near the site of injury. These macrophages have a positive roles at the injury site after PNI (Cofano et al., 2019). Macrophages, as with other inflammatory cells, are attracted to damaged tissue and play a vital role in regulating the inflammatory, proliferation, and regeneration of the tissue’s injured during the inflammatory process. Among inflammatory cells, macrophages demonstrate both pro-inflammatory (M1) and anti-inflammatory (M2) effects. In essence, M1 macrophages kickstart the healing process in the initial three to 5 days by clearing debris and pathogenic contamination through phagocytosis and secreting TNFα, IL-1α and IL-1β and metalloproteinase (Liu et al., 2019). Subsequently, the transition of M1 macrophages to M2 macrophages and the release of anti-inflammatory cytokines like IL-4/IL-13 and IL-10 promote activities such as proliferation, maturation, migration, resolution of inflammation, and angiogenesis (Boukelmoune et al., 2021; Sharifi et al., 2024).

MSC secretion can also exert immunomodulatory, anti-inflammatory, neurotrophic, neuroprotective, and angiogenic effects on the host microenvironment. MSCs can contribute to PN regeneration by providing an enhanced neuroprotective microenvironment that prevents neurodegeneration and apoptosis while supporting neurogenesis, axonal growth, remyelination, and cell metabolism (Widgerow et al., 2013).

With the secretion of VEGF, MSCs have neurotrophic and mitogenic effects on peripheral nerves. In addition, the MSCs secretome induces axonal growth and Schwann cell proliferation following trauma. Finally, MSCs can also promote the proliferation and survival of neurons by inhibiting inflammatory responses and pro-apoptotic pathways, which represents critical steps for inducing nerve regeneration (Wang et al., 2019).

Various synergistic strategies can be observed in PN regeneration, with the most common ones involving the synergistic effect of MSCs and chemotherapy, cell therapy, conduits, nanomaterials, and stimulators. In all of these cases, the impact of MSCs on the regenerative activity of PN can be assessed in two ways: protection and regeneration (Laroni et al., 2015; Volkman and Offen, 2017). The protective effects of MSCs usually involve cell secretions that modulate immune-inflammatory functions, optimize the environment by reducing oxidative stress, strengthen neural structures, prevent abnormal tissue formation, and extend the lifespan of neurons (Li et al., 2022). MSCs’ regenerative action is more focused on their ability to differentiate into neurons or induce the differentiation of neural progenitor cells into neurons (Lo Furno et al., 2018). Despite the success and promising results achieved by synergistic strategies in MSC-based PN regeneration after injury, the mechanisms underlying the protective and regenerative effects of MSCs in these strategies remain unclear and contradictory.

### 3.1 Synergistic effect of MSCs and therapeutic compounds in repair of neuropathy and inflammation

#### 3.1.1 Neuropathy

**FIGURE 3:**
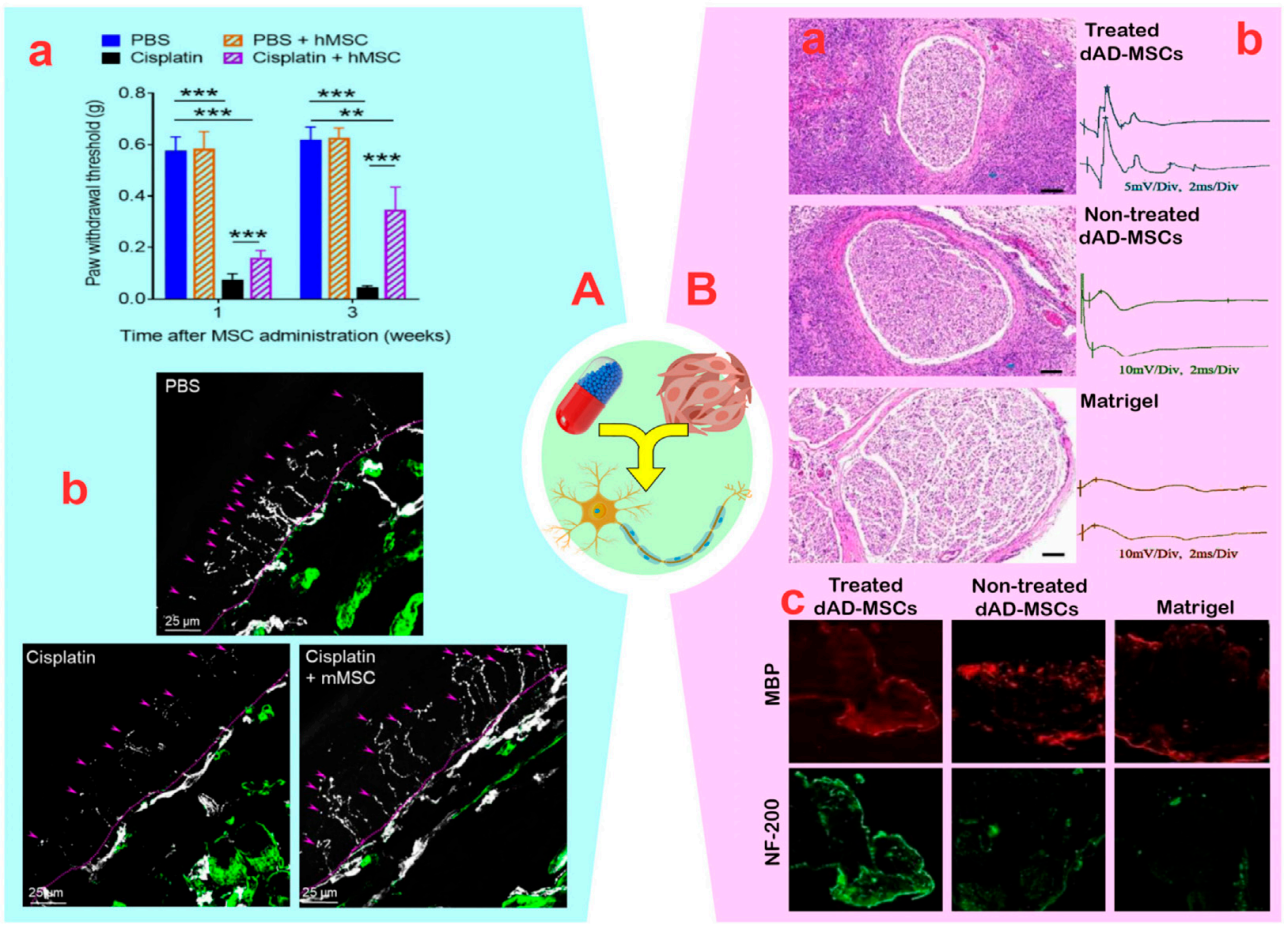
**(A)**: **(A)** Mice were treated with two cycles (48 and 96 h) of cisplatin (2.3 mg/kg/day); after the last cisplatin dose, 1×10^6^ human mesenchymal stem cells (MSCs) was administered via the nasal route. Mechanical allodynia was measured using von Frey hairs (**p < 0.01 and ***p < 0.001). **(B)** human MSCs administration reverses the loss of intra-epidermal nerve fibers in the hind paw of cisplatin-treated mice. The basement membrane is indicated by the dashed lines, nerve fibers crossing the basement membrane are indicated by arrows. Reprinted with permission from ref. (Boukelmoune et al., 2021). **(B)**: **(A)** Histological evaluation of regenerated nerves at 3 weeks after cell transplantation. **(B)** Representative oscillograms of each group at 3-week post-surgery. **(C)** The immunofluorescence photographs of the myelinated nerve fibers and the regenerated axons. Myelin basic protein (MBP) and NF-H Antibody (NF)-200 positive axons were stained with red and green fluorescence. Reprinted with permission from ref. (Yao et al., 2021).

It is crucial to protect the peripheral nerves of cancer and diabetes patients from neuropathy caused by harmful drugs and biological agents. Neuropathic damage is frequently the result of oxidative stress, mitochondrial damage, cell death, changes in ion channel activity, microtubule damage, axonal degeneration, and demyelination (Martini and Willison, 2016). MSCs seem to have the potential to address these issues by reducing inflammation, promoting the activation of progenitor cells and neuronal differentiation, and optimizing the environment. In this regard, Mannelli et al. (2018) found that synergizing adipose-derived MSCS (AD-MSCs, 2 × 10^6^) with oxaliplatin (2.4 mg/kg) effectively manages chemotherapy-induced neuropathic pain in colorectal cancer in a rat model. The reduction of neuropathic pain was attributed to the reversal of increased VEGF-A levels and a decrease in the amount of the VEGF165b isoform caused by AD-MSCs (Mannelli et al., 2018). However, using AD-MSCs presents challenges due to its limited distribution to non-target tissues and lower analgesic efficacy compared to the anti-VEGF-A monoclonal antibody bevacizumab (15 mg/kg). In another study, Al-Massri et al. (2019) found that combining BM-MSCs (1 × 10^6^) with pregabalin (30 mg/kg) reduced the negative impact of paclitaxel on the sciatic nerve as compared to using either approach alone. They discovered that this combined approach increased the total antioxidant capacity content by 1.34–1.48 times and the nerve growth factor (NGF) content by approximately ∼1.2-fold compared to using each method separately. Additionally, the combined use of BM-MSCs and pregabalin led to a further decrease in the expression of genes encoding the NF-kB p65 (∼1.8-fold), TNF-α (∼2.1-fold), and IL-6 (∼2.5-fold) compared to using pregabalin alone. Furthermore, the co-administration of BM-MSCs and pregabalin resulted in a reduction in inflammation through a decrease in the protein expression of phosphorylated p38 mitogen-activated protein kinase from ∼3.9 to ∼0.9 (AU) and caspase-3 from ∼14 to ∼4.9 (ng/mg) in the injured sciatic nerve. Subsequently, an increased axon count, optimized myelination, and improved sensory-motor function in rats confirmed the regenerative and anti-inflammatory effects of co-administering BM-MSCs and pregabalin compared to a singular approach (Al-Massri et al., 2019). Another study discovered that the administration of BM-MSCs with cisplatin in cancer treatment increased IL-10 levels produced by macrophages, significantly reducing pain and paw harms caused by neuropathy (Figure 3A) (Boukelmoune et al., 2021). While the rate of PNI healing depends on factors such as drug dosage, prescribed compounds, injury site state, treatment duration, and wound location, Sezer et al. (2022) demonstrated that increasing the number of BM-MSCs from 1 × 10^6^ to 5 × 10^6^ in mice with paclitaxel-induced neuropathy reduced the healing time of the sciatic nerve from 30 to 15 days. MSCs have clinical applications, but the distribution of cells to non-target tissues, determination of cell number, and the balance of drug dose:cell number for PN regeneration pose major challenges.

Diabetic neuropathy, much like chemotherapy neuropathy, is influenced by the combined action of MSCs or their secretions with therapeutic substances. The condition involves demyelination of peripheral nerves and dysfunction of nerve fibers due to oxidative stress induced by high blood sugar levels in neurons. Diabetes worsens the degeneration of the peripheral nervous system by diminishing the transmission of brain-derived neurotrophic factor (BDNF), NGF, and neurotrophin-3 in peripheral nerves, as well as reducing the secretion of insulin-like growth factors (Sezer et al., 2022). In a study by Abdelrahman et al. (2018), it was demonstrated that the combined effect of BM-MSCs and fluoxetine enhanced BDNF, VEGF, and IL-10 in a model of diabetic neuropathy induced by streptozocin, particularly at concentrations above 2 μM of fluoxetine. The researchers observed an increase in paracrine secretion and a significant reduction in the effects of neuropathy. The positive impact of the synergy between BM-MSCs and fluoxetine on neuropathy treatment is further supported by the improved structure of the sciatic nerve, including increased perineurium thickness with collagen, enhanced vascularity, absence of neurogenic edema, improved myelination, and the falciform nucleus of Schwann cells (Abdelrahman et al., 2018).

#### 3.1.2 Inflammation Guardian

Using immune system modulators such as dexamethasone and tacrolimus can significantly impact peripheral nerve repair (Uzun et al., 2019). For example, Moattari et al. (2018) found that combining umbilical cord MSCs (UC-MSCs) (300,000 cells) and dexamethasone (1 mg/kg) within a polymer membrane increased nerve conduction velocity from 4 mV to 5 mV and improved the sciatic function index (SFI) (−60.41). This synergistic effect led to a significant increase in neuron number, improved nerve fiber diameter, and complete myelination of the transected sciatic nerve, compared to using dexamethasone and MSCs alone (Moattari et al., 2018). Additionally, another study described that the synergistic effect of AD-MSCs with tacrolimus not only improved cell survival during PNI repair without cytotoxic effects (Saffari T. M. et al., 2021), but also enhanced sciatic nerve myelination and neurite length (from 10% to 22%) (Saffari S. et al., 2021). In a study by Saffari S. et al. (2021), the synergistic effect of AD-MSCs with tacrolimus in autologous nerve tissue transplantation was found to be more effective than the use of allograft in sciatic nerve transplantation. Although this study examined the myelination ability by AD-MSCs, the absence of investigation into neurotrophic function makes analysis difficult. In a subsequent study, Yao et al. (2021) reported that the combination of AD-MSCs with tacrolimus resulted in significant improvements in PNI. This synergistic effect not only increased the neuron length from 120 to 200 μm compared to using a single method, but also significantly enhanced the secretion of neurotrophic factors. The qRT-PCR results showed that the combined effect of AD-MSCs with tacrolimus significantly increased the expression of BDNF, glial-derived growth factor (GDNF), and NGF genes, particularly at concentrations ranging from 1 to 10 ng/mL. Additionally, in animal models, the synergism of AD-MSCs and tacrolimus led to improvements in the SFI (−74.62 to −41.66), nerve conduction velocity, muscle mobility, and muscle fiber area (Figure 3B). There was also a positive effect on the diameter of nerve fibers, which increased from 2.4 to 4 µm. Overall, despite the relative success of neuropathy treatment through synergistic effects, numerous concerns remain regarding MSCs migration, cell or drug dosage, tumor safety, response degree, MSC distribution, transplant rejection, potential of patient-derived MSCs, and lack of clarity in anti-inflammatory and regenerative mechanisms.

### 3.2 Synergistic effect of MSCs and derivatives of nerve cells in PNI repair

Regeneration after PNI typically requires the activation of neural stem or progenitor cells from the niche. However, obstacles like low cell viability and inadequate proliferation hinder full repair of peripheral nerves. MSCs offer a promising solution for enhancing regenerative processes thanks to their capacity to differentiate into neurons, regulate the immune system, and stimulate growth and proliferation through paracrine secretion. Despite the various functions of MSCs, two approaches are favored to examine relationships between MSCs and neural progenitor cells: (1) cell-cell contact and (2) effect of vesicular secretions.

#### 3.2.1 Cell-cell contact

**FIGURE 4:**
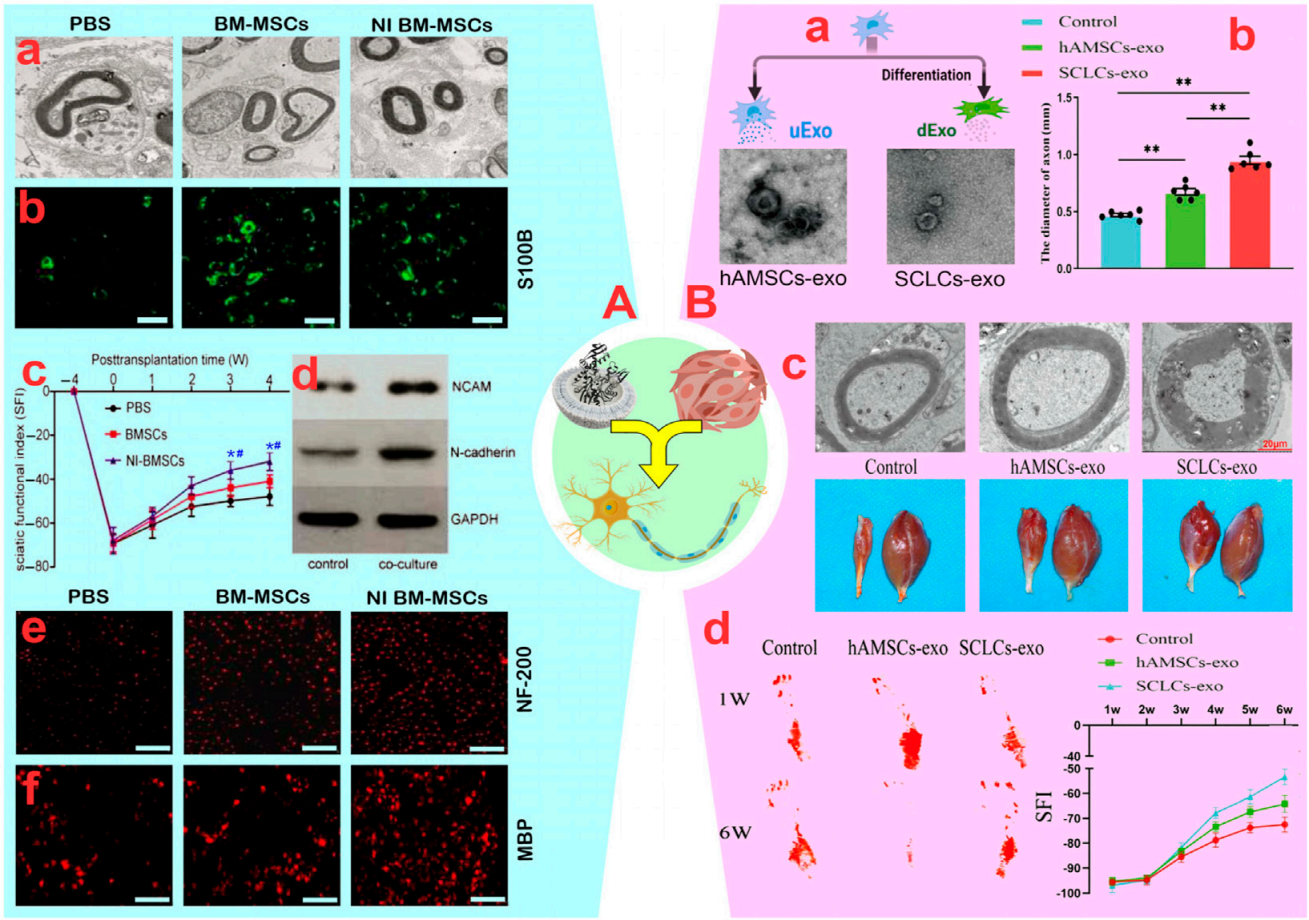
**(A)**: **(A)** The ultrastructure of native denervated Schwann cells observed in the different groups. **(B)** Neuron-induced (NI) bone marrow-mesenchymal stem cells (BM-MSCs) promoted proliferation of native Schwann cell based on S100β (green) staining (scale bar: 200 µm). **(C)** Functional recovery of the sciatic nerve. *p < 0.05, vs. PBS group; #*p* < 0.05, vs. BM-MSCs group. **(D)** The expression of NCAM and N-cadherin in Schwann cells increased significantly after they were co-cultured for 48 h **(E, F)** Increased Myelin basic protein (MBP) and NF-H Antibody (NF)-200 in co-cultures stained for axonal regeneration and myelination (scale bar: 200 µm). Reprinted with permission from ref. (Zheng et al., 2018). **(B)**: **(A)** Schematic view of the generation and analysis of exosomes. **(B)** Diameter of the regenerated nerves of the rats in groups (**p < 0.01). **(C)** Top: Representative TEM images of sciatic nerves in rats, and Bottom: observation of hind limb gastrocnemius muscle in rats. **(D)** Footprints of rats in each group at weeks one and six post-surgery and sciatic function index (SFI) values of the rats in groups. Reprinted with permission from ref. (Hu et al., 2023).

While the use of MSCs presents challenges such as its distribution to non-target tissues, their direct use is appealing because of their ability to modulate the immune system, secrete neurotrophic factors, and alter neuronal phenotypes. For instance, Marconi et al. (2012) modulated the immune system and enhanced sciatic nerve regeneration by systemically injecting AD-MSCs in combination with Schwann cells. AD-MSCs raised GDNF and IGF-I levels, sustained BDNF levels, and enhanced Schwann cell survival, proliferation, and differentiation. AD-MSCs also improved the regeneration of crushed sciatic nerves by encouraging nerve fiber sprouting and increasing the number of nerve fibers by about 40%, as indicated by higher levels of GAP-43 (a marker of axonal regeneration). Furthermore, there was an increase in fiber length and a notable reduction in the number of monocytes, macrophages, and CD3 lymphocytes, all of which aid in axonal regeneration. AD-MSCs injection significantly improved SFI, plantar flexion, and toe extension compared to control mice after 21 days (Marconi et al., 2012). However, the systemic administration of AD-MSCs has been challenging due to their high concentrations in lymphoid organs and limited presence in inflamed PNIs. Zheng et al. (2018) demonstrated that co-administration of BM-MSCs and Schwann cells led to significant improvements in the SFI, number of innervated axons, G ratio, myelination, and number of Schwann cells in the sciatic nerve (Figure 4A). They also observed that inducible BM-MSCs (iBM-MSCs) generated in neural medium containing inactive Schwann cells reactivated Schwann cells after injury. This reactivation by iBM-MSCs resulted in a more pronounced increase in SFI, axon number, G ratio, and myelination rate compared to the control. Additionally, the increased secretion of neurotrophic factors such as BDNF, NGF, and nortrophin-3, and the enhancement of NCAM and N-cadherin by iBM-MSCs (Figure 4A) improved the sciatic nerve regeneration rate and enhanced cell adhesion (Zheng et al., 2018). In another study, it was found that increasing the secretion of BDNF and NGF, along with PC12-TrkB using lentivirus-engineered BM-MSCs, induced significant neurite outgrowth in each neuron (Uz et al., 2020).

#### 3.2.2 Vesicular secretions

Despite issues such as non-target distribution of MSCs, tumorigenicity, and so on, the incomplete penetration of MSCs to neural tissues due to epineurium–endoneurium blockage poses challenges to systemic injection. Therefore, utilizing MSC-secreted vesicles containing various compounds in cooperation with neurons is an appealing alternative. In a study by Mao et al. (2019), the development of sciatic nerve axons and myelination was stimulated through the synergistic effect of G-MSC-derived vesicles (103.8 nm), as confirmed by increased tubulin-3, protein expression of GFAP and EGR2/KROX-20, and improved neuromuscular junction (NMJ). Furthermore, enhancements in gastrocnemius muscle weight, SFI, paw expansion, and footprint validated the synergistic impact of G-MSC-derived vesicles in sciatic nerve regeneration. This data suggests a comparable synergistic function between G-MSC-derived vesicles with neural progenitor cells, similar to direct injection of G-MSCs (Mao et al., 2019). In the next study, the authors found that the synergy of G-MSC-derived exosomes (102 nm) and chitin-based conduits increased the proliferation of Schwann cells and dorsal root ganglion (DRGs) in 10 mm sciatic nerve defects (Rao et al., 2019). In fact, the synergistic effect of exosomes and chitin-based conduits doubled the axon length, nerve fiber number and diameter, and myelin membrane size over 12 weeks. This improvement in the gastrocnemius muscle, muscle structure, and sensory-motor indicators highlights positive synergistic effects (Rao et al., 2019). Contrary to these results, Bucan et al. (2019) demonstrated that co-administration of AD-MSCs with neurons in a damaged environment enhanced the regeneration process compared to AD-MSC-derived exosomes alone. This was achieved through an increase in the number of nerve branches per neuron (∼115 vs. ∼17) and an increase in the length of neurites per neuron (∼117.4 vs. ∼72.9 µm) (Bucan et al., 2019). Variations in the content of exosomes, especially neurotrophic factors, and secretion levels are likely the main factors contributing to these differences. For instance, exosomes (∼141 nm) from inducible AD-MSCs (iAD-MSCs) produced in neural medium exhibited a stronger combined impact with Schwann cells in comparison to AD-MSCs for neural repair (Liu et al., 2022). The introduction of iAD-MSC-derived exosomes containing miRNA-22-3p into neurospheres inhibited phosphatase and tensin expression and AKT/mTOR activation, resulting in Schwann cell proliferation and migration as well as longitudinal axon growth. Additionally, in contrast to AD-MSC-derived exosomes, iAD-MSC-derived exosomes demonstrated notably reduced TNF, IL-6, IL-1B, NF-κB, and NO_2_ levels, thereby aiding the repair process through inflammation reduction (Liu et al., 2022). Also, Schwann cell-like cell-derived (SCLC) exosomes (30–150 nm) differentiated from amniotic-derived MSCs (AM-MSCs) exhibited increased expression of GDNF, NGF, MBP, SOX10, and Oct-6 genes compared to AM-MSC-derived exosomes (Hu et al., 2023). Consequently, this led to a higher density of myelinated nerve fibers, thicker myelin membrane, and increased weight of the gastrocnemius muscle in the injured sciatic nerve (Figure 4B).

Ma et al. (2019) enhanced sciatic nerve regeneration by decreasing inflammation through the collaboration of human UC-MSCs-derived vesicles in the 80–650 nm range with Schwann cells. They found that the vesicles have a crucial role in facilitating neuronal regeneration in the distal nerve stump by reducing pro-inflammatory cytokines (IL-6 and IL-1β) and increasing the expression of the anti-inflammatory cytokine IL-10. The beneficial effects of this synergy were validated by enhanced myelination (based on S-100 and NF-200 markers), strengthened gastrocnemius muscle, increased axon count, and improved movement patterns in mice (Ma et al., 2019). These findings have clearly demonstrated the therapeutic potential of injured Schwann cells induced by UC-MSCs vesicles. The synergistic effects of human UC-MSC-derived exosomes with olfactory ensheathing cells (OECs) tripled the number of OECs in the hypoxic environment of injured sciatic nerves, raising hopes for therapy (Zhang Y. et al., 2020). Zhang et al. (2020b) showed that the released exosomes effectively regulated the migration of OECs in a hypoxic environment and enhanced cell proliferation and differentiation by increasing gene expression of BDNF and other neurotrophic factors. In the rat model, the use of human UC-MSC-derived exosomes led to an optimal distribution of Schwann cells, improved axon regeneration, increased SFI, and nerve conduction velocity.

### 3.3 Synergistic effect of MSCs and conduits in PNI repair

#### 3.3.1 Biological conduits

Several biological pathways, such as arteries, veins, muscles, amniotic membrane, and neural trunks, have been developed and extensively utilized for PN regeneration in a relatively brief timespan. While the utilization of biological pathways, including neural and vascular grafts, has proven to be highly effective, this approach is only successful for short-term regeneration due to the rapid degradation of biological conduits into inert materials (Houshyar et al., 2019). Furthermore, the constraints of allograft systems have prompted a shift in focus towards the utilization of allograft systems, despite the inflammation linked to graft rejection.

##### 3.3.1.1 DNTA

Due to limited resources, multiple surgeries, and sensory-motor issues associated with autologous nerve grafting for nerve defects with gaps larger than 10 mm, the use of DNTA is considered a viable alternative. DNTA can facilitate the regeneration process by providing internal structural and extracellular matrix components (Hopf et al., 2022). Conflicting results are generally attributed to incomplete decellularization, cell type loading, and decellularization solvents. In a rat model of sciatic nerve transection (10–15 mm), Zhao et al. (2011) and Wang et al. (2012) showed that the synergistic effect of DNTA with 5 × 10^5^ and 1 × 10^6^ BM-MSCs, respectively, decreased inflammation, increased axon length, and gained triceps weight. Zhao et al. (2011) reported increased myelin thickness and improved SFI, which differed from the results of Wang et al. (2012). Furthermore, it was recognized that there was no significant difference between the synergistic effect of DNTA and BM-MSCs or AD-MSCs in regenerating injured sciatic nerves (Zhao et al., 2011). Despite these successes, there is no guarantee that MSCs will convert into neurons, nor is there conclusive evidence about their efficiency in calling neurons. For example, despite the significant synergistic effect of AD-MSCs with DNTA on increasing the number of neurons in the transected sciatic nerve (10 mm), the survival rate of AD-MSCs gradually decreased at the second week, based on the decrease in bioluminescence signal from 6.28 × 10^4^ to 3.73 × 10^4^ (Rbia et al., 2019b). Additionally, the absence of migration of AD-MSCs into adjacent tissues and their removal at day 29 indicates the instability of MSCs in the long-term process of sciatic nerve regeneration (Rbia et al., 2019b).

**FIGURE 5:**
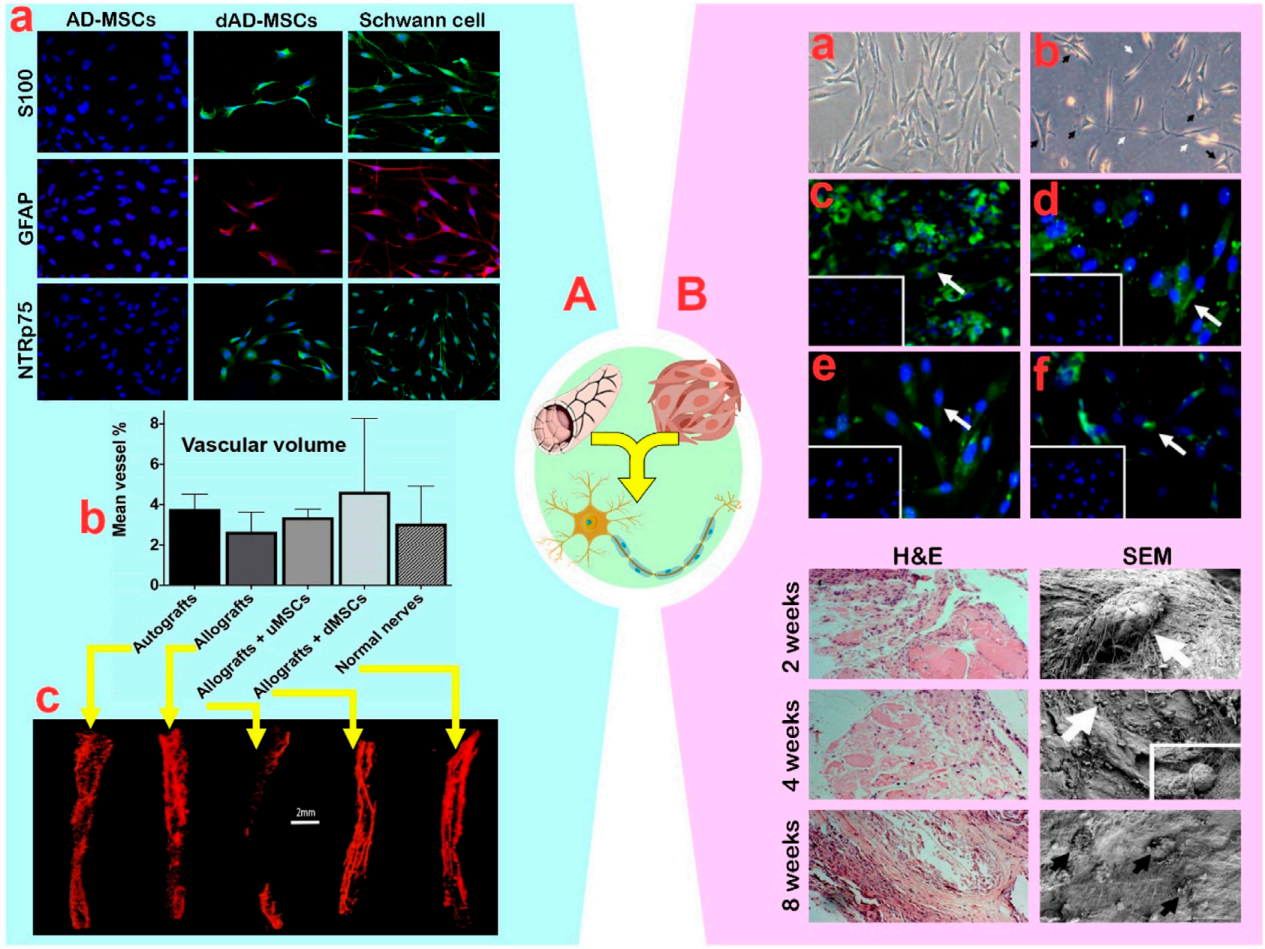
**(A)**: **(A)** Confirmation of adipose-mesenchymal stem cells (AD-MSCs) differentiation by expression of Schwann cell markers S100, GFAP, and NTR p75. **(B)** The vascular volume outcomes of different groups with undifferentiated AD-MSCs and differentiated AD-MSCs. **(C)** The obtained micro-CT scans that served for the volume measurements of normal veins in nerves. Reprinted with permission from ref. (Mathot et al., 2020a). **(B)**: **(A)** Undifferentiated bone marrow (BM)-MSCs displayed a flat fibroblast-like morphology with a spindle shape. **(B)** After induction, the differentiated BM-MSCs finally changed into star shaped-cells (black arrows) with elongated processes (white arrows). **(C–F)** Differentiated MSCs expressed the Schwann cell surface markers S100b, GFAP, nestin, and p75NGF receptor, respectively. The insert exhibits that the undifferentiated BM-MSCs were negative for Schwann cell markers. Down plots: Hematoxylin and eosin and SEM analysis of muscle stuffed vein-based conduits at 2, 4, and 8 weeks post implantation. Seeded cells were producing new matrix (white arrows). Matured cells within a dense homogenous matrix (black arrows). Reprinted with permission from ref. (Hassan et al., 2012).

A common hypothesis about the role of MSCs in the PN regeneration process is that they do not convert into neurons, but rather enhance paracrine secretions to optimize the environment. Rbia et al. (2019a) reported that the synergistic effect of AD-MSCs and DNTA improved nerve fiber number, angiogenesis, and myelination through a significant increase in neurotrophic factors (BDNF, PTN, GAP43), angiogenic agents (VEGF, PECAM), and myelination factors (MBP, MPZ, PMP22). These molecular changes indicate a positive potential for the synergy between MSCs and DNTA in PN regeneration. Another study showed that the paracrine secretions of Schwann-like cells arising from AD-MSCs are similar to that of AD-MSCs in terms of synergistic activity with DNTA (Mathot et al., 2020b). The profiles and secretion rates of neurotrophic factors (NGF, GDNF, GAP-43), cell cycle regulator (CCNB2), and angiogenic agent (VEGF1) in differentiated AD-MSCs and AD-MSCs were different during the first 14 days, but the secretion levels after 21 days were not different. In confirmation of this finding, the study by Mathot et al. (2020a) showed that the synergism of DNTA with Schwann-like cells differentiated from AD-MSCs increased sciatic nerve angiogenesis from 29.2% to 38.9% (Figure 5A). Nonetheless, the ultimate vascular volume of both groups remained unchanged.

##### 3.3.1.2 DBV

DBV-based conduits, whether arterial or venous, are receiving more attention than DNTA-based conduits due to their higher durability, slower degradation, lower cost, and higher flexibility. The integration of macro-, micro-, and nano-structures into DBV ducts and their capacity to interact with the extracellular matrix, prevent luminal collapse, reduce neuromas, and minimize potential scarring, have unexpectedly made the use of DBV-based conduits advantageous (Kaizawa et al., 2017; Gontika et al., 2018). In this regard, Liao et al. (2013) discovered that while the pain reflexes and sensory-motor patterns of mice in the DNTA and DBV groups were similar, the DBV’s structure with more elastic fibers prevents faster conduit collapse during long-term repairs. In a study by Sun et al. (2011b), the synergistic effect of AD-MSCs with DBV improved unilateral vibrissae movement and nerve fiber number after 8 weeks compared to using either method alone. Although superior axonal growth and improved innervation were observed, the synergistic effect of AD-MSCs with DBV had no significant effect on myelin membrane thickness compared to either method used individually (Sun et al., 2011b).

In confirmation of the above findings, the synergistic effect of AD-MSCs (1 × 10^4^) and DBV did not impact myelin thickness or G ratio, despite improving the lag time and increasing the diameter of myelinated nerve fibers (Sun et al., 2011a). While the synergistic impact on neural tissue regeneration is significant, the lack of attention to the fate of AD-MSCs and their conversion efficiency into neuron-like cells hinders detailed analysis. Although the differentiation of BM-MSCs into neuron-like cells led to an increase in GFAP (up to 75%), S100β (up to 45%), nestin (up to 35%) and NGF (up to 30%) markers (Hassan et al., 2012), the precise reason for the high differentiation efficiency of MSCs into stable neuron-like cells remains unclear. Nevertheless, Hassan et al. (2012) demonstrated that transplanting differentiated BM-MSCs (3.0 × 10^6^) into DBV and then into the injured sciatic nerve resulted in enhanced neuronal proliferation and the formation of new matrix from DBV-based conduits over an 8-week period (Figure 5B). Furthermore, the degradation of DBV-based conduits for neural tube recovery did not cause inflammation in neural tissue (Hassan et al., 2012). In the following study, it was reported that the synergism of differentiated BM-MSCs (3.0 × 10^6^) with DBV decreased the autotomy behavior of mice and traumatic neuroma in mice (Ramli et al., 2019). Similar to the previous findings, the G-ratio (0.77 vs. 0.59), fiber diameter (3.09 vs. 2.55), and axon diameter (2.34 vs. 1.39) were not found to have a significant impact (Ramli et al., 2019). It appears that extending the treatment duration from short (<8 weeks) to moderate (8–12 weeks) provides ample time for neural progenitor cells to emerge, effectively reducing the reported abnormalities. Moreover, using allogeneic MSCs instead of xenogeneic MSCs enhances the healing process. For instance, in a transected rat sciatic nerve model, the synergism of murine AD-MSCs (1 × 10^6^) with DBV as an allograft, compared to canine AD-MSCs (1 × 10^6^) as a xenograft, led to improved SFI (−53.58 vs. −86.60), latency (1.75 vs. 3.1 m/s), and amplitude value (9.76 vs. 3.23) (Sanchez et al., 2017). The results indicated that allogeneic AD-MSCs were comparable with the positive control group and superior to other groups. However, the relative superiority of canine AD-MSCs over murine AD-MSCs in fiber density, number, and increased expression of BDNF and S100β was significant (Sanchez et al., 2017).

#### 3.3.2 Polymer-based conduits

The excellent performance of polymer-based conduits in peripheral nerve regeneration, achieved through micro- and nano-structures and the ability to transport active molecules or drugs, has led to their widespread use (Pinho et al., 2016). Polymer-based conduits are particularly attractive for therapeutic interventions in peripheral nerves due to their high efficiency in 1–2 cm gaps, abundant availability, long-term stability due to physicochemical properties, ease of preparation, and low cost (Jiang et al., 2020). The polymer used, whether natural or synthetic, must be non-toxic, non-immunogenic, permeable, flexible, electrically conductive if possible, and capable of degrading into by-products at an appropriate rate. The biological properties of a conduit are influenced by its chemical properties, molecular weight, construction technique, and loading, which should not adversely affect the biological interaction of the conduits with cells (Zhang et al., 2022).

##### 3.3.2.1 Natural Polymers

**FIGURE 6:**
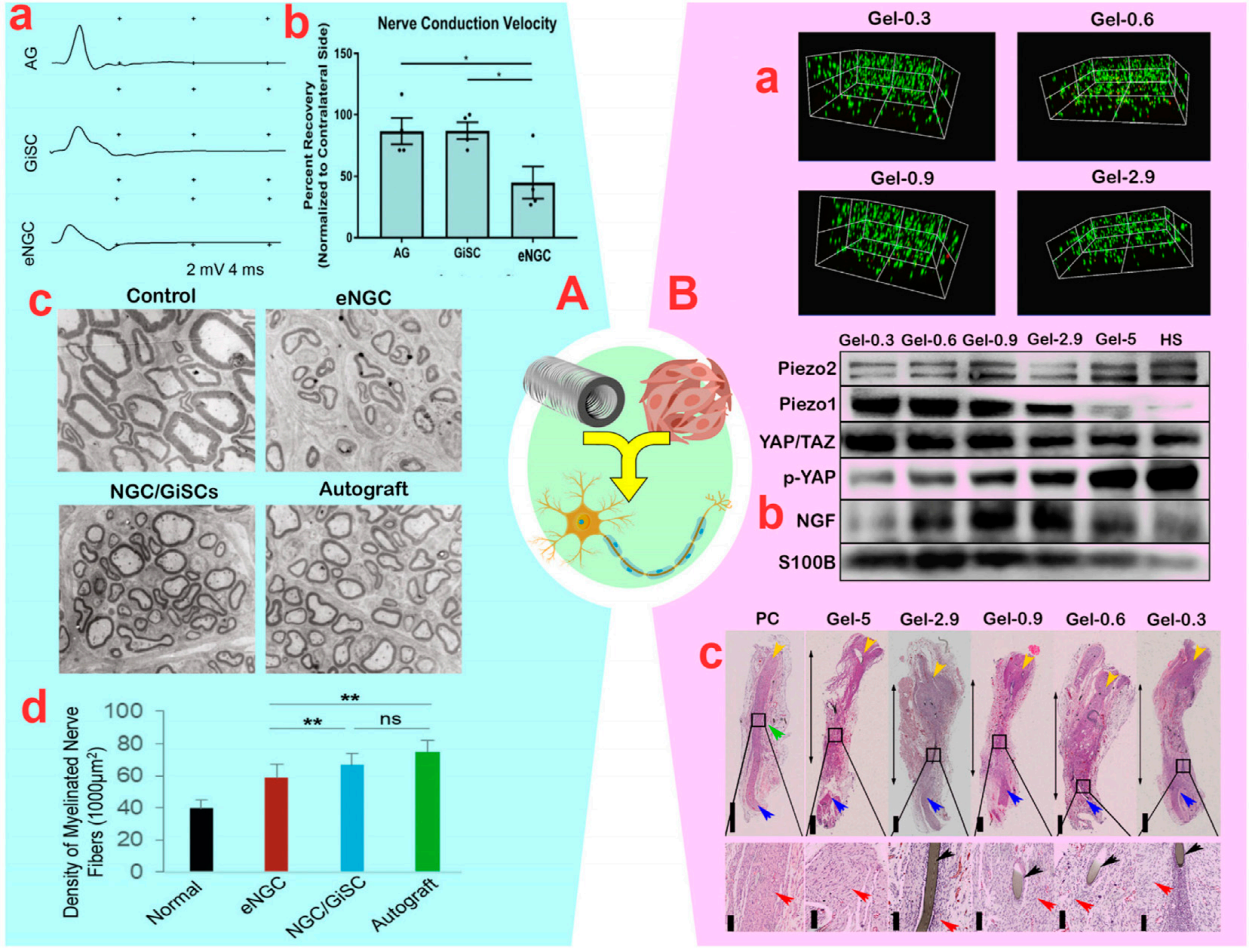
**(A)**: **(A)** Compound muscle action potential recordings of the vibrissal muscles of rats in empty nerve guide conduits (eNGC), nerve autografts (AG), or NGC laden with gingiva-mesenchymal stem cells (G-MSCs) (*p < 0.05). **(B)** Motor nerve conduction velocity of rats. **(C)** Transmission electron microscopy of ultrathin sections of the newly regenerated facial nerve. **(D)** Quantification of the density of myelinated axons (the number of myelinated axons/1,000 μm^2^) (ns: non-significant and **p < 0.01). Reprinted with permission from ref. (Zhang Q. et al., 2021). **(B)**: **(A)** Survival assay of bone marrow (BM)-MSCs encapsulated in GelMA (Gelatin Methacrylate) hydrogels. **(B)** Immunoblotted image for PIEZO2, PIEZO1, YAP/TAZ, p-YAP, GFAP, NGF, S100b in BM-MSCs plated on GelMA after co-culture with NE-4C. **(C)** Hematoxylin and eosin staining of sciatic nerves (double-headed arrows: the regenerated nerves; yellow arrows: the proximal end; blue arrows: the distal end; black arrows: the residual stitch; red arrows: the renascent nerve fibers; green arrow: enlargement site of the regenerated nerve). Reprinted with permission from ref. (Gao et al., 2023).

Despite the potential toxicity of by-products from the degradation of natural polymers, the advanced control of inflammation by MSCs will make the use of these materials less problematic. In this context, Ladak et al. (2011) studied the synergistic effect of retinoic acid-treated BM-MSCs (0.8 × 10^6^) with a collagen-based conduit, significantly improving sciatic nerve regeneration by increasing neurite length and motor neuron number, without causing inflammation or toxicity. Although the regenerative capacity of BM-MSCs with the collagen-based conduit was lower than that of autologous transplants, the presence of differentiated BM-MSCs within the conduit resulted in more motor neurons and neuron-like cells compared to empty conduits. Despite the success of PN regeneration, the moderate efficiency (51%) of converting BM-MSCs into neuron-like cells based on the expression of 51% GFAP, 47% S100, and 45% NGFR in the regenerative pathway remains a challenge with the number of neuron cells (Ladak et al., 2011). To address the challenge of loaded cell number, it is crucial to use conduits with aligned fibers. Cui et al. (2018) developed collagen-based conduits with longitudinally aligned fibers and synergized them with differentiated UC-MSCs, without causing inflammation or toxicity. This approach facilitated sciatic nerve regeneration in dogs and enhanced their performance. The improvements in muscle function, reduced latency, and increased gastrocnemius muscle weight demonstrate the remarkable impact of the synergistic effect of UC-MSCs and collagen-based conduits. Further evidence of the synergistic effects were significant increases in fiber diameter, G ratio, and myelin thickness by up to 4-fold, as well as increases in the expression of S100 (50%), NF (30%), and GAP-43 (80%), respectively (Cui et al., 2018). In another study, Zhang Q. et al. (2021) indicated that the synergistic effect of G-MSCs (2 × 10^6^) and collagen-based conduits led to a higher quantity of myelinated axons, enhanced myelin sheath thickness, increased nerve conduction velocity, and improved muscle action potentials, ultimately resulting in more effective PN regeneration (Figure 6A). While the combined use of MSCs and collagen-based conduits has demonstrated positive results in PN regeneration, the rapid degradation and low mechanical strength of collagen make its use challenging. Chitosan has longer stability and higher strength than collagen, making chitosan-based conduits suitable for longer-term treatments with larger gaps. For instance, the synergy between (10^7^) BM-MSCs and a chitosan-based conduit (acetylation level: 95%) was found to yield comparable positive results to autografts in the regeneration of severed sciatic nerves, without causing inflammation or toxicity (Zheng and Cui, 2012). This synergized approach notably enhanced the SFI, fiber density, and fiber diameter after 6 weeks, exceeding the results of individual methods. Extending the treatment period to 12 weeks, Zhu et al. (2015) showed that the synergistic effect of BM-MSCs and chitosan-based conduits enhanced the repair of transected sciatic nerves more effectively than individual approaches, as evidenced by increased SFI (−62.83 vs. −81.67), muscle action potential (∼50.63 vs. ∼38.23 mV), and nerve conduction velocity (∼21.9 vs. ∼19.12 m/s). Morphological parameters also showed improvement, including increased myelin sheath thickness (0.59 ± 0.13 vs. 0.31 ± 0.13 µm), fiber diameter (3.90 ± 0.94 vs. 2.96 ± 1.24 µm), and number of motor neurons (9.11 ± 1.64 vs. 6.67 ± 1.89) (Zhu et al., 2015), indicating a more promising regeneration process compared to the 6-week treatment period. Recently, Alvites et al. (2021) demonstrated that Olfactory Mucosa-MSCs (1 × 10^6^) synergized with a chitosan-based conduit improved sensory-motor parameters (SF, SS, WR, and kinetics) and morphological characteristics (fiber area, fiber density, myelin thickness, G-ratio, and axon diameter) of the neural tissue in a transected sciatic nerve (15 mm) compared with individual approaches. This improvement was similar to autografts and did not cause adhesion, pain, or neurotmesis.

##### 3.3.2.2 Synthetic Polymers

The impurity, unclear connections and anchor points, and relatively weak mechanical properties of natural polymers have led researchers to consider synthetic polymers (Gregory and Phillips, 2021). Despite the existence of many different types of synthetic polymers, polycaprolactone (PCL) is the most commonly used synthetic polymer in conduit construction due to its high processability, excellent compatibility, good mechanical, facile processability and acceptable permeability, and topographical properties, and long-term degradability (due to its five hydrophobic–CH2 moieties) (Zhang X. et al., 2020). Hence, employing PCL polymer in lengthy nerve conduits with gaps exceeding 15 mm has become prevalent. The channels should possess ample strength to facilitate the development of regenerated nerves over an extended duration while degrading *in vivo* at a suitable pace. Nonetheless, given the hydrophobic nature of PCL surfaces, the application of water-compatible coatings is crucial. Frattini et al. (2012) showed that the synergistic effect of BM-MSCs interacting with PCL-based conduits results in an increase in the quantity of myelinated fibers, the number of neurons in the DRG, and an upregulation of Schwann cell signaling markers (S100 and GFP) compared to individual methods. Substantial increases in BDNF (2-fold), NGF (3-fold), and neurotrophin-4 (2.5-fold) secretions, as well as improvements in the gastrocnemius muscle weight, SFI, and alkaline phosphatase levels within 6 weeks, confirmed the positive effects of BM-MSCs on sciatic nerve regeneration in the PCL-based conduit. Another study found that the combined effect of BM-MSCs and PCL-based conduits increased the number of myelinated fibers and G ratio in neurons with a diameter >0.7 μm, thereby restoring the function of transected S1 nerves within 10 weeks (Oliveira et al., 2014). While there were no significant differences in fiber diameter, axonal diameter, or myelin thickness, the structure of the fascicles improved compared to a single approach, and the synergistic effect enhanced the rat paw’s response (Oliveira et al., 2014). Despite the positive feedback from the above studies, the fate and location of MSCs or progenitor neurons in PCL-based conduits remains uncertain. In this field, Carrier-Ruiz et al. (2015) discovered that the BM-MSCs nuclei spread over the PCL protrusions and the cell bodies extended into grooves/fibers in polyethylene-based conduits covered with PCL. This approach seems to effectively protect neurons by expanding the available biological surface area and creating a feeder layer. The rise in Schwann cell calling and a 90% increase in survival rate is consistent with these events. Additionally, they discovered that the synergistic effect of BM-MSCs and a conduit led to greater neural tissue thickness, increased neuron count, axonal density, locomotor index (footprint area, maximum step intensity, and swing speed), more organized axons, and re-innervation of the motor plate compared to individual methods (Carrier-Ruiz et al., 2015). In another study, alignment of PCL fibers in conduits, instead of random fibers, notably enhanced Schwann cell migration, just as a gradient of BM-MSC-derived neurotrophic factors facilitated migration and development (Sun et al., 2019). Consequently, the synergism of conduits containing aligned fibers with BM-MSCs enhances the migration of neural progenitor cells and the speed of peripheral nerve regeneration. The increased SFI, longitudinal growth of nerve fibers, and proportion of myelinated axons validate the favorable effects of this synergism (Sun et al., 2019). Recently, Entezari et al. (2022) demonstrated that olfactory ecto-derived MSCs (OE-MSCs) differentiate into Schwann cells more effectively in the 3D space of conduits compared to the 2D space. Additionally, the incorporation of PPy polymer into the PCL lumen promoted the differentiation of OE-MSCs into Schwann cells, as confirmed by the upregulation S100, p75, and MBP markers. While the length of PC12-derived neurons increased in PCL-PPy-based conduits containing OE-MSCs, the synergistic effects did not impact nerve conduction velocity, cell adhesion, proliferation rate, survival, or distribution of cells. Previous studies showed that adding PPy to PVA-based conduits and its synergy with UC-MSCs increased SFI and axonal diameter without affecting cell adhesion or viability (Ribeiro et al., 2015). This indicates that UC-MSCs anti-inflammatory secretions can prevent PPy-induced inflammation. Rodríguez-Sánchez et al. (2021) demonstrated that the synergistic effect of AD-MSCs, in PCL-based conduits, improved SFI and increased myelin fibers and sheaths by promoting the neurotrophic factors BDNF, GDNF, and HGF. MSCs enhance neuronal function within conduits by increasing IL-10 and reducing inflammation (Rodríguez-Sánchez et al., 2021). While MSCs have demonstrated promising results in anti-inflammatory and neuroregeneration-inducing effects, the function of MSC-derived Schwann-like cells remains unclear. In this context, Gao et al. (2023) demonstrated that raising the gelatin strength in GelMA-based conduits from 0.3 to 2.9 kPa decreased the migration of BM-MSCs into the environment and facilitated their differentiation into Schwann cells through a synergistic approach (Figure 6B). In fact, it was observed that YAP/TAZ mechanical signaling in the nucleus of BM-MSCs increased with PIEZO1 expression (growth pathways for differentiation), when the conduit strength decreased from five to 0.3 kPa. This finding is consistent with the increased differentiation of BM-MSCs into Schwann-like cells, as indicated by higher levels of S100β and NGF in the strength range from 2.9 to 0.9 kPa (Figure 6B). Meanwhile, prolonged co-culture of BM-MSCs with NE-4C in synergism with GelMA-based conduits featuring aligned fibers induced morphological alterations in BM-MSCs, resulting in a spindle-shaped morphology and elongated protrusions. Enhancements in the SFI, muscle action potential, and thermal response, along with improvements in muscle fiber diameter, vascular density, number of myelinated axons, and axon diameter, collectively indicate the beneficial synergy between BM-MSCs and GelMA-based conduits (Gao et al., 2023). Although Schwann cell-derived BM-MSCs have shown robust success in PN regeneration, recent evidence supports the positive synergistic effect of BM-MSCs-based vesicles with conduits in promoting nerve sprouts formation in PN regeneration (Zhang et al., 2023).

### 3.4 Synergistic effect of MSCs and NPs in PNI repair

#### 3.4.1 Organic NPs

During nerve regeneration, organic NPs such as micelles, liposomes, vesicles, dendrimers, nanofibers, and carbon nanomaterials are increasingly used. The synergism of MSCs with vesicles, nanofibers, and carbon nanomaterials has garnered the most attention for PN regeneration. Exosomes, which are nanometer-sized vesicles, play crucial roles in cell-cell interactions (Zheng et al., 2020). The secretion of exosomes from Schwann cells during nerve injury and their impact on neural tissue regeneration suggests the potential of exosomes in the differentiation of MSCs into neuron-like cells (Yu et al., 2021). Wang et al. (2020) showed that the combined effect of BM-MSCs with 50–80 nm exosomes from RSC96 altered the shape of BM-MSCs from spherical to spindle-shaped. Cell differentiation was confirmed by an increase in nerve fiber length-to-width ratio and elevated expression of Schwann cell-specific markers including genes encoding S100, GFAP, Sox10, NGFR, and EGR2 (Wang et al., 2020). Likewise, after extracting 127.5 ± 2.1 nm exosomes from RSC96, the synergistic effect of AD-MSCs with RSC96 exosomes not only altered cell morphology, but also enhanced the expression of S100β, NGFR, MPZ, and GFAP markers in differentiated cells (Zhou et al., 2022). RSC96 exosomes control the differentiation of AD-MSCs into Schwann cells via the PIK3CD and p-Akt pathways. However, the mechanism of MSC differentiation through Schwann cell-derived exosomes remains unclear and requires further investigation.

**FIGURE 7:**
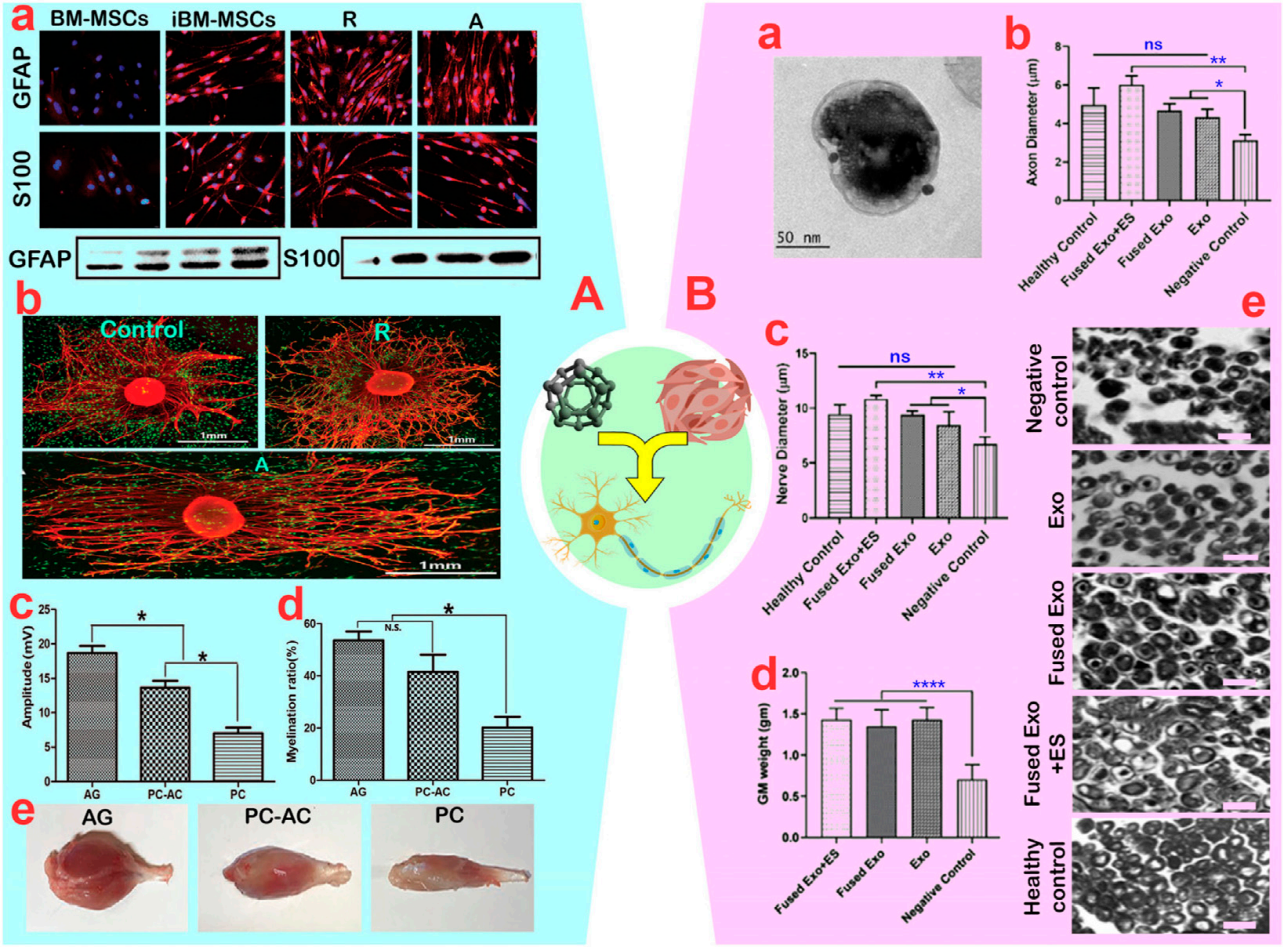
**(A)**: **(A)** Green fluorescent protein-bone marrow-mesenchymal stem cells (GFP-BM-MSCs) induced on aligned **(A)** and random (R) nanofibers with quantitation of GFAP and S100 protein levels. **(B)** Demonstration of neurite outgrowth from dorsal root ganglion (DRG) with induced BM-MSCs co-culture on nanofibers. **(C, D)** Quantitative analysis of the amplitude and myelination rate of axons in AG (autograft), PC-AC (Polycaprolactone containing BM-MSCs graft) and PC (Polycaprolactone conduit) (ns: non-significant and *p < 0.05). **(E)** A view of the gastrocnemius muscle in rats. Reprinted with permission from ref (Hu et al., 2020). **(B)**: **(A)** TEM image of exosomes from BM-MSCs. Nerve morphometric analysis in the groups for **(B)** axon diameter and **(C)** nerve diameter. (ns: non-significant, *p < 0.05 and **p < 0.01). **(D)** quantification of the muscle weight from the treated hind limb (****p < 0.0001). **(E)** Histopathology of isolated sciatic nerve samples showing improvement in the tissue organization in the treated groups (scale bar: 10 µm). Reprinted with permission from ref. (Singh et al., 2021).

Carbon nanomaterials are being considered for nerve repair due to their high electrical conductivity, nano-topological properties, mechanical strength, and flexibility. Combining MSCs with carbon nanomaterials significantly reduced the inflammatory effects of carbon, making them widely usable. After reducing the collagen hydrogel’s electrical resistance by CNTs (30 nm in diameter and hundreds of nanometers in length), Lee et al. (2014) found that the synergism of BM-MSCs with CNTs in collagen hydrogel led to elevated levels of the neurotrophic factors GAP-43 (3–4 times), NGF (10–15 times), and BDNF (9–12 times) secreted from BM-MSCs. Additionally, CNTs affected the regeneration process by boosting BM-MSC proliferation and changing cell morphology to an elongated and oriented state. The enhanced performance of BM-MSCs in synergy with CNTs (0.5%–1%) resulted in increased neurite growth, a threefold rise in neurite length, and a 2–3-fold increase in neurite-containing cells (Lee et al., 2014). Ribeiro et al. (2015) demonstrated that the synergistic effect of UC-MSCs with PVA-CNT-based conduits enhanced the SFI, fiber density, number of nerve fibers, myelin membrane thickness, and the percentage of area in the severed sciatic nerve. The synergistic effects of UC-MSCs with PVA-CNT-based conduits reduced muscle weight and muscle fiber area, which is inconsistent with the results for motor parameters and neuronal cell structure. The diminished muscle tissue regeneration in this synergy was attributed to an excessive rise in calcium and magnesium levels, which is the downside of synergy (Ribeiro et al., 2015). Following this, PCL-gelatin-based conduits with amine-functionalized CNT fibers, in comparison to random conduits, not only enhanced the growth of BM-MSCs and DRG cells, but also promoted the differentiation of BM-MSCs into Schwann-like cells, as evidenced by the increase in S100 and GFAP markers (Figure 7A) (Hu et al., 2020). Moreover, regardless of the organization and randomness of the CNT fibers within the conduit, it was shown that the synergistic effect of BM-MSCs with PCL-gelatin-based conduits containing aligned CNTs increased the number of axons, myelination rate, nerve conduction velocity, and muscle action potential (Figure 7A) (Hu et al., 2020). In another study, it was revealed that the synergistic effect of AD-MSCs with poly (p-dioxanone) NYs-based conduits containing CNTs promoted the growth/proliferation of Schwann cells along with the differentiation of AD-MSCs into Schwann-like cells based on S100 and MBP markers (Wu et al., 2022). Furthermore, the increased expression of myelination markers such as S100, NGFR, MBP, and MPZ in Schwann cells, along with the enhanced release of neurotrophic factors such as NGF, HGF, and EGF, indicates a beneficial synergistic effect in the nerve repair process (Wu et al., 2022). Similar to CNTs, graphene oxide has been shown to facilitate nerve regeneration by enhancing cell adhesion strength through its physicochemical and topographical properties. For instance, Llewellyn et al. (2021) showed that the synergistic effect of AD-MSCs with graphene oxide resulted in increased proliferation and differentiation of AD-MSCs into Schwann-like cells with spindle-shaped morphology. However, despite the increased NGF protein production *in vitro*, this synergistic effect did not lead to improved nerve regeneration *in vivo* due to the lack of impact on NGF protein production. Confirming this finding, it was determined that the co-administration of AD-MSCs and graphene oxide did not have a significant impact on the sprouting rate, despite an increase in the length of DRG neurites. NGF plays a crucial role in the regeneration and sprouting of axons in primary sensory neurons (Fornaro et al., 2020).

#### 3.4.2 Inorganic NPs

Although many inorganic NPs have been created for regenerative purposes, only a small number have been used for peripheral nerve regeneration. Generally used NPs for detecting and repairing peripheral nerves are metal and alloy NPs, silica nanostructures, and magnetic NPs. To our knowledge, iron oxide (IO) and gold (Au) NPs are commonly used in synergy with MSCs. Karimi et al. (2021) showed that the synergistic effect of OE-MSCs and IONPs (10 ± 2 nm) in alginate fibers changed the morphology of OE-MSCs from round to spindle-shaped with longer fibers compared to alginate nanofibers or alginate hydrogels. The mechanism of these morphological changes remains unclear, but it seems that the presence of nano-sized protrusions by IONPs promotes cell cytoplasmic expansion and differentiation into Schwann-like cells by enhancing cell adhesion. Additionally, the decrease in nestin, corresponding to greater cell proliferation (Bagher et al., 2018), and the increase in β-tubulin-3 and GFAP supports the positive effect of IONPs in synergy with OE-MSCs to generate Schwann-like cells (Karimi et al., 2021). Despite the dose-dependent toxicity of IONPs, it was discovered that the synergistic effect actually enhanced the viability of OE-MSCs by up to 10 mg/mL IONPs when compared to alginate hydrogels and alginate nanofibers. Meanwhile, based on FDA data, IONPs concentrations up to 24 mg/mL did not have any negative effects on UC-MSCs activity (Hu et al., 2009). Consequently, the accumulation of IONPs-binding agent, particularly glutaraldehyde, on alginate is likely to induce toxicity and apoptosis at higher IONPs concentrations. Subsequently, Ghaderinejad et al. (2021) demonstrated that the synergistic effect of OE-MSCs with IONPs in aligned alginate fibers enhanced the proliferation (up to 20%) and differentiation of OE-MSCs into Schwann-like cells (based on a 2-fold increase of tubulin-3 and GFAP). Additionally, they observed that altering the fiber orientation from random to oriented successfully reduced the toxicity of IONPs (Ghaderinejad et al., 2021). Another study utilizing a rat model of sciatic nerve injury showed that the synergistic effect of AD-MSCs with IONPs (3–16 nm) effectively preserved myelinated axons, increased myelin membrane thickness, improved amplitude, enhanced muscle action potential, and decreased latency compared to individual approaches (Soto et al., 2021). Increased levels of tubulin three and MBP confirmed the beneficial synergistic effect of AD-MSCs with IONPs on sciatic nerve regeneration. Despite the positive influence of IONPs on the conversion of AD-MSCs into Schwann-like cells, the accumulation of IONPs-labeled AD-MSCs in targeted tissue exceeded that of AD-MSCs due to the magnetic field. This non-invasive approach accelerates the pace of regeneration in injured sciatic nerves.

Due to the lower toxicity and higher electrical conductivity of AuNPs compared to IONPs, there is interest in using AuNPs for PN regeneration. In a rat model of transected sciatic nerves, the synergistic effect of BM-MSCs (1 × 10^7^) with PCL-based conduits containing 1% polydopamine-coated AuNPs (15 mm) increased the expression of S100, nestin, NF-200, and Tuj1 neurofilaments 200 as axonal specific markers (Qian et al., 2018). This result demonstrates that the synergistic effect of BM-MSCs and polydopamine-AuNPs/PCLs-conduits has a positive impact on cell growth and differentiation. Additionally, the presence of AuNPs within the conduits increased the size of the F-actin cytoskeleton and cell adhesion. In polydopamine-AuNP/PCL-based conduits, BM-MSCs and Schwann cells showed increased spindle-shape structure and provided more axons. The improved SFI, muscle function potential, number of myelinated axons, and thickness of myelin sheaths, nerve conduction velocity, and angiogenesis over 18 weeks indicate a beneficial synergistic effect in PN regeneration.

### 3.5 Synergistic effect of MSCs and electrical stimulation in PNI repair

Research has indicated that the electrical stimulation of peripheral nerves, particularly at low frequencies, results in enhanced nerve regeneration and improved function, irrespective of the length of axonal damage and the slowness of their growth (Zuo et al., 2020). While the synergistic effect of electrical stimulation with MSCs has shown promise in animal studies for nerve regeneration (Ashour et al., 2015), its potential application in human clinical practice is still uncertain. Nevertheless, the use of electrical stimulation instead of chemical/cellular stimulation is remarkable due to the elimination of chemical processing, cost-effectiveness, control over the spatio-temporal differentiation of cells, and the formation of neural circuits. Das et al. (2017) demonstrated that the combined impact of electrical stimulation (100 mV signal with 50 Hz frequency, 10 min per day) with BM-MSCs on graphene substrates notably enhanced the paracrine secretion of NGF, GDNF, and BDNF similar to chemical stimulation (mercaptoethanol, retinoic acid, forskolin, and heregulin). Furthermore, a positive effect of electrical stimulation similar to chemical stimulation is validated through the increase in p75, S100, and S100β markers (Das et al., 2017). These findings are consistent with those of Uz et al. (2020), who also illustrated that the synergistic effect of electrical stimulation with BDNF-transfected BM-MSCs on graphene substrates amplified NGF and GDNF production, as well as the expression of Schwann cell-specific markers (S100, S100β, p75, MAP, and Tuj1). NGF secretion increased to 50 ng/mL when the voltage was changed from 25 to 100 mA at 50 Hz. However, this voltage change did not affect the specific markers S100, S100β, and p75. When PC12-TrkB was cultured with BDNF-transfected BM-MSCs under electrical stimulation, the neurite length increased by 1.5–2-fold and neuronal myelination by 90% (Uz et al., 2020). Similarly, Uz et al. (2019) demonstrated that the synergistic effect of electrical stimulation (100 mV at 50 Hz, 2 min per day) with BM-MSCs on graphene/gelatin-based conduits increased NGF secretion and induced differentiation of BM-MSCs into Schwann-like cells, as evidenced by increased expression of p75, S100, and S100β markers. The application of gelatin to graphene, along with the presence of BM-MSCs, not only prevented the expected toxicity of graphene to neurons, but also led to the formation of 3D intracellular networks within the lumen through the induction of cell proliferation (Uz et al., 2019). Cell differentiation on carbon nanomaterial-containing conduits seems to be affected by focal adhesion kinase, amplification of the mitogen-activated protein kinase (p38) signaling pathway, alteration of cell membrane potential, regulation of ion channels, activation of calcium channels, and regulation of depolarization. However, a challenge in utilizing carbon sheets for transmitting electrical impulses is the potential increase in dedifferentiation of differentiated MSCs.

Kim et al. (2020) enhanced the length of neurites in BM-MSCs by using AuNPs-polyaniline polymer nanospheres (180 ± 20 nm) and combining them with electrical stimulation. This resulted in neurites reaching up to 170 μm, a significant increase compared to the group without NPs and individual approaches. Through microscopy and specific markers MAP2 and Tuj1, they also observed a change in the phenotype of BM-MSCs into Schwann-like cells due to this synergistic effect. The mechanism of action involves a potential 10% increase in electrical conductivity by NPs and an impact on Ca^2+^ influx into the cytoplasm to stimulate protein kinase C via the ERK1/2 pathway. Singh et al. (2021) recently demonstrated in a diabetic neuropathy model that the synergy approach of internalizing PPy-NPs (88.40 ± 3.46 nm) into BM-MSC-derived exosomes (211.8 ± 76.5 nm) with electrical stimulation (2 Hz frequency and 1 mA) enhanced the diameter of nerve fibers and axons in the sciatic nerve compared to individual treatments (Figure 7B). This resulted in a notable increase in nerve conduction velocity to 57.60 ± 0.45 m/s and a rise in muscle action potential to 16.96 ± 0.73 mV, indicating a positive synergistic effect of the treatment process. While there were no significant effects on myelin sheath thickness, axon density, muscle structure, or gastrocnemius muscle weight, the elevation of S100, MBP, and MPZ markers suggested a favorable synergistic effect on axon regeneration.

## 4 Clinical application

Since the first clinical studies on the use of MSCs in hematological malignancies in 1995, research has been conducted on the use of MSCs for treating or regenerating various diseases (Guillamat-Prats, 2021). Numerous clinical studies based on reports from www.ClinicalTrials.org have been conducted for the treatment of neurological diseases. Despite promising experimental results, the use of synergistic approaches in clinical applications has been delayed due to the contradictory results. Although various licenses have been granted for the independent use of conduits, NPs, cell therapy, drug delivery, and electrical stimulation to repair neural tissue (Table 3), the use of synergistic approaches remains debated. Lack of complete knowledge about immune system responses, unexpected behavior of neurons and MSCs, and ethical or legal issues are the main reasons for the slow development of synergistic approaches in clinical work.

**TABLE 3:** Summary of clinical application of different therapeutic approaches in PN regeneration.

Subject under investigation	Conditions	Interventions	NCT number
Safety and efficacy of autologous Schwann cell augmentation in severe peripheral nerve injury	Peripheral nerve injury	Biological: Autologous human Schwann cell	NCT05541250
Safety of cultured allogeneic UC-MSCs for trigeminal neuralgia and peripheral neuropathy	Peripheral neuropathy Trigeminal neuralgia	Biological: AlloRx	NCT05152368
Human amniotic membrane and MSCs composite	Brachial plexus neuropathies	Procedure: Nerve transfer procedure Procedure: Nerve transfer with AD-MSCs composite wrapping	NCT04654286
A novel synthetic polymer nerve conduit ‘polynerve’ in participants with sensory digital nerve injury	Injury of nerves at wrist and hand level	Device: Polynerve	NCT02970864
Nerve repair using hydrophilic polymers to promote immediate fusion of severed axons and swift return of function	Peripheral nerve injury	Drug: Polyethylene glycol	NCT02359825
Mid-term effect observation of biodegradable conduit small gap tublization repairing peripheral nerve injury	Peripheral nerve injury	Other: Degradable conduit small gap tublization	NCT03359330
Reconstruction of digital nerve lesions with muscle-in-vein conduits	Peripheral nerve injury upper limb	Other: Nerve reconstruction	NCT04788030
A comparative post-marketing study of commercially available peripheral nerve gap repair options	Traumatic nerve injury	Device: Hollow tube nerve conduits, synthetic or biosynthetic	NCT00948025
Promoting healing of injured nerves with electrical stimulation therapy	Peripheral nerve injury Peripheral nerve injury upper limb	Device: Checkpoint BEST System	NCT05884125
Electrical stimulation to enhance peripheral nerve regeneration	Peripheral nerve injury	Procedure: Post-surgical electrical stimulation	NCT02403661
The effect of pre-operative electrical stimulation on peripheral nerve regeneration.	Peripheral nerve injury Sensory deficit Digital nerve lesion	Procedure: Electrical stimulation Procedure: Sham stimulation	NCT03205124
Registry of Avance^®^ Nerve Graft’s utilization and recovery outcomes post peripheral nerve reconstruction	Peripheral nerve injury	Other: Processed human nerve graft Other: Standard treatment, autogenous nerve graft, direct sutureetc.	NCT01526681
Tesamorelin to improve functional outcomes after peripheral nerve injury	Peripheral nerve injury	Drug: Tesamorelin 2 Milligrams Drug: Placebo	NCT03150511

## 5 Challenges and future perspectives

Studies have shown promising results using MSCs to regenerate peripheral nerves in synergy with therapeutic techniques such as drug therapy, cell therapy, and electrical stimulation (Zuo et al., 2020; Supra et al., 2023; Sharifi et al., 2024). However, technical challenges make it difficult to implement synergistic approaches. Many MSC-based regenerative products are not FDA-approved, making it difficult to transition MSCs from the laboratory to clinical use. The biggest challenges are:

Standardization: Despite the effectiveness of regeneration methods in research, there is no specific standard for the type and number of MSCs, cell cycle state, culture media, transfer time to damaged site, and minimum time required to regenerate peripheral nerve functions (Ikebe and Suzuki, 2014). Although the type of treatment, type of PNI, and person`s lifestyle can affect MSC activity, the lack of convergence in these cases makes it difficult to determine the level of influence of MSCs.

Evaluation index: While metrics for evaluating neural tissue like morphology, structure, and sensory-motor function are valid, they cannot definitively determine the impact of MSCs on neural tissue regeneration (Lavorato et al., 2021). This is because the current criteria do not allow for independent observation of MSCs during long-term regeneration processes, including migration, half-life, heterogeneous differentiation, MSC-derived neurofibrillary function, degree of integrity, and various protective-regenerative functions. In addition to focusing on labeling and technical analysis of lab-on-a-chip or organoids, altering the regeneration concept or optimizing the regeneration process based on the presence of MSCs can partially address the challenges of regeneration with MSCs.

Pathological events: Although MSCs have demonstrated significant therapeutic potential in regenerative processes, the issue of whether MSCs can contribute to tumorigenesis, fibrosis, and acute inflammation remains unanswered (Li et al., 2019). Given that MSCs, similar to other stem cells, have the ability to differentiate into cell types other than Schwann-like cells or undergo de-differentiation, the occurrence of such biological phenomena is not unexpected. Additionally, MSCs produce cytokines, such as chemokines and growth factors, which can directly stimulate cancer cell receptors and support tumor growth. Therefore, it is important to evaluate both tumorigenic and regenerative indicators to assess potential cancer risk before starting clinical trials.

Analytical models: One of the major challenges in medical research is the critical incompatibility between *in vitro*, animal, and human models (Sharifi et al., 2022a). Studies on the protective and regenerative potential of MSCs *in vitro* are generally uncertain. They do not replicate *in vivo* biological interactions such as cell migration, adhesion, proliferation, growth, differentiation, and their associated secretions. Additionally, differences in immune systems, regeneration rates, and expansion of damaged tissue between animal models and humans can bias results (Ribitsch et al., 2020). Focusing on large animals and using new techniques such as tissue printing and lab-on-a-chip with human cells could help solve this problem.

Commercialization: MSCs are typically cultured in flasks and specific quantities for research. However, large-scale industrial production makes it challenging to control MSCs quality, leading to non-targeted mutations (Jankovic et al., 2023). Prolonged culture time and increased passage number can result in cell aging, negatively impacting regeneration activity once the Hayflick number is reached. Consequently, commercial or large-scale expansion may face challenges with cell aging and mutations across multiple passages. Presently, the use of MSC-derived exosomes, along with exclusive cultures of recipient cells, is seen as a therapeutic solution, raising questions about the adoption of a standard protocol.

## 6 Conclusion

Apart from the gold standard of the autologous graft, a reliable strategy has not yet been established despite the existence of various treatment methods such as allograft transplantation, drug therapy, cell therapy, and electrical stimulation. The PN regenerative process is inherently dynamic and requires more flexible treatments to be multifunctional and controllable. Among various approaches, the synergistic effect of MSCs with macro, micro, and nano strategies to control inflammation and provide neural progenitor cells is surprising. MSCs effectively control inflammation, recruit nerve progenitor cells, promote neuronal proliferation and growth through the secretion of neurotrophic factors, and differentiate into Schwann-like cells. Treatment strategies depend on the type and severity of the injury. The results show that synergizing MSCs with biological and pharmaceutical compounds is commonly used for diabetic neurological disorders or chemotherapy-induced damage. Repairing PNI with moderate to large gaps is typically done through the combination of MSCs with biological and polymeric conduits. However, non-invasive treatment may use synergistic effect of electrical stimulation with the injection of MSCs or their exosomes, particularly for the reconstruction of PNIs with gaps less than 4 mm. However, its clinical use is limited due to uncertainties in the following challenges: achieving ideal conversion of MSCs into Schwann-like cells accompanied with neural function, dedifferentiation of MSC-derived nerve cells, preventing tumorigenesis, ensuring the stability of MSCs in the damaged site, and avoiding immune responses. Another important issue with synergistic approaches is the lack of consistent and reproducible results that can be related to MSCs source, technique, and type of injury. Despite the aforementioned challenges, experimental evidence indicates that the combination of MSCs and therapeutics can enhance neural regeneration. However, additional research is necessary to translate therapeutic potential into treatment gains for clinical use.

## Abbreviations

AD-MSCs: Adipose-derived mesenchymal stem cells, AM-MSCs: Amniotic-derived mesenchymal stem cells, BDNF: Brain derived neurotrophic factor, BM-MSCs: Bone marrow-derived mesenchymal stem cells, CNTs: Carbon nanotubes, DBV: Decellularized blood vessels, DNTA: Decellularized nerve trunk of allografts, DRG: Dorsal root ganglion, GAP-46: Growth-associated protein 46, GelMA: gelatin methacrylate, GDNF: Glial-derived neurotrophic factor, GFAP: Glial fibrillary acidic protein, G-MSC: Gingiva-derived mesenchymal stem cells, H&E: hematoxylin and eosin, IGF-I: Insulin-like growth Factor-I, IL: Interleukin, MBP: myelin basic protein, MSCs: Mesenchymal stem cells, NCC: Neural crest cell, NF: Neurofilament, NF-κB: Nuclear factor kappa B, NGF: nerve growth factor, NPs: Nanoparticles, OE-MSCs: Olfactory ecto-derived mesenchymal stem cells, PCL: Polycaprolactone, PNI: Peripheral nerve injury, PPy: Polypyrrole, PVA: Polyvinyl alcohol, SFI: Sciatic function index, TNF-α: Tumor necrosis factor-alpha, UC-MSCs: Umbilical cord-derived mesenchymal stem cells, VEGF: Vascular Endothelial Cell Growth Factor.

SMARTQ

## References

[B1] Abdelrahman S. A. Samak M. A. Shalaby S. M. (2018). Fluoxetine pretreatment enhances neurogenic, angiogenic and immunomodulatory effects of MSCs on experimentally induced diabetic neuropathy. Cell tissue Res. 374, 83–97. 10.1007/s00441-018-2838-6 29687216

[B2] Al-Massri K. F. Ahmed L. A. El-Abhar H. S. (2019). Mesenchymal stem cells therapy enhances the efficacy of pregabalin and prevents its motor impairment in paclitaxel-induced neuropathy in rats: role of Notch1 receptor and JAK/STAT signaling pathway. Behav. Brain Res. 360, 303–311. 10.1016/j.bbr.2018.12.013 30543902

[B3] Alvites R. D. Branquinho M. V. Sousa A. C. Amorim I. Magalhães R. João F. (2021). Combined use of chitosan and olfactory mucosa mesenchymal stem/stromal cells to promote peripheral nerve regeneration *in vivo* . Stem cells Int. 2021, 1–32. 10.1155/2021/6613029 PMC7801080 33488738

[B4] Ashour F. A. Elbaz A. A. Sabek N. A. Hazzaa S. M. Metwally E. M. (2015). Effect of electrical stimulation and stem cells on experimentally induced peripheral nerve injury in rats. Menoufia Med. J. 28 (3), 742. 10.4103/1110-2098.167896

[B5] Bagher Z. Kamrava S. K. Alizadeh R. Farhadi M. Absalan M. Falah M. (2018). Differentiation of neural crest stem cells from nasal mucosa into motor neuron-like cells. J. Chem. Neuroanat. 92, 35–40. 10.1016/j.jchemneu.2018.05.003 29807106

[B6] Berebichez-Fridman R. Montero-Olvera P. R. (2018). Sources and clinical applications of mesenchymal stem cells: state-of-the-art review. Sultan Qaboos Univ. Med. J. 18 (3), e264. 10.18295/squmj.2018.18.03.002 30607265 PMC6307657

[B7] Boukelmoune N. Laumet G. Tang Y. Ma J. Mahant I. Singh S. K. (2021). Nasal administration of mesenchymal stem cells reverses chemotherapy-induced peripheral neuropathy in mice. Brain, Behav. Immun. 93, 43–54. 10.1016/j.bbi.2020.12.011 33316379 PMC8826497

[B8] Bucan V. Vaslaitis D. Peck C.-T. Strauß S. Vogt P. M. Radtke C. (2019). Effect of exosomes from rat adipose-derived mesenchymal stem cells on neurite outgrowth and sciatic nerve regeneration after crush injury. Mol. Neurobiol. 56, 1812–1824. 10.1007/s12035-018-1172-z 29931510 PMC6394792

[B9] Carrier-Ruiz A. Evaristo-Mendonça F. Mendez-Otero R. Ribeiro-Resende V. (2015). Biological behavior of mesenchymal stem cells on poly-ε-caprolactone filaments and a strategy for tissue engineering of segments of the peripheral nerves. Stem Cell Res. Ther. 6 (1), 128. 10.1186/s13287-015-0121-2 26149068 PMC4522087

[B10] Chan K. M. Gordon T. Zochodne D. W. Power H. A. (2014). Improving peripheral nerve regeneration: from molecular mechanisms to potential therapeutic targets. Exp. Neurol. 261, 826–835. 10.1016/j.expneurol.2014.09.006 25220611

[B11] Charbord P. (2010). Bone marrow mesenchymal stem cells: historical overview and concepts. Hum. gene Ther. 21 (9), 1045–1056. 10.1089/hum.2010.115 20565251 PMC4823383

[B12] Cofano F. Boido M. Monticelli M. Zenga F. Ducati A. Vercelli A. (2019). Mesenchymal stem cells for spinal cord injury: current options, limitations, and future of cell therapy. Int. J. Mol. Sci. 20 (11), 2698. 10.3390/ijms20112698 31159345 PMC6600381

[B13] Cui Y. Yao Y. Zhao Y. Xiao Z. Cao Z. Han S. (2018). Functional collagen conduits combined with human mesenchymal stem cells promote regeneration after sciatic nerve transection in dogs. J. Tissue Eng. Regen. Med. 12 (5), 1285–1296. 10.1002/term.2660 29499096

[B14] Das S. R. Uz M. Ding S. Lentner M. T. Hondred J. A. Cargill A. A. (2017). Electrical differentiation of mesenchymal stem cells into Schwann‐cell‐like phenotypes using inkjet‐printed graphene circuits. Adv. Healthc. Mater. 6 (7), 1601087. 10.1002/adhm.201601087 28218474

[B15] Entezari M. Mozafari M. Bakhtiyari M. Moradi F. Bagher Z. Soleimani M. (2022). Three-dimensional-printed polycaprolactone/polypyrrole conducting scaffolds for differentiation of human olfactory ecto-mesenchymal stem cells into Schwann cell-like phenotypes and promotion of neurite outgrowth. J. Biomed. Mater. Res. Part A 110 (5), 1134–1146. 10.1002/jbm.a.37361 35075781

[B16] Fornaro M. Giovannelli A. Foggetti A. Muratori L. Geuna S. Novajra G. (2020). Role of neurotrophic factors in enhancing linear axonal growth of ganglionic sensory neurons *in vitro* . Neural Regen. Res. 15 (9), 1732. 10.4103/1673-5374.276338 32209780 PMC7437584

[B17] Frattini F. Pereira Lopes F. R. Almeida F. M. Rodrigues R. F. Boldrini L. C. Tomaz M. A. (2012). Mesenchymal stem cells in a polycaprolactone conduit promote sciatic nerve regeneration and sensory neuron survival after nerve injury. Tissue Eng. Part A 18 (19-20), 2030–2039. 10.1089/ten.tea.2011.0496 22646222

[B18] Gao S. Tang Y. Sun W. Liu Z. Zhao T. Li X. (2023). 3D-bioprinted GelMA nerve guidance conduits promoted peripheral nerve regeneration by inducing trans-differentiation of MSCs into SCLCs via PIEZO1/YAP axis. Mater. Today Adv. 17, 100325. 10.1016/j.mtadv.2022.100325

[B19] Ghaderinejad P. Najmoddin N. Bagher Z. Saeed M. Karimi S. Simorgh S. (2021). An injectable anisotropic alginate hydrogel containing oriented fibers for nerve tissue engineering. Chem. Eng. J. 420, 130465. 10.1016/j.cej.2021.130465

[B20] Gontika I. Katsimpoulas M. Antoniou E. Kostakis A. Stavropoulos-Giokas C. Michalopoulos E. (2018). Decellularized human umbilical artery used as nerve conduit. Bioengineering 5 (4), 100. 10.3390/bioengineering5040100 30469361 PMC6315692

[B21] Gregory H. Phillips J. B. (2021). Materials for peripheral nerve repair constructs: natural proteins or synthetic polymers? Neurochem. Int. 143, 104953. 10.1016/j.neuint.2020.104953 33388359

[B22] Gu Y. Li Z. Huang J. Wang H. Gu X. Gu J. (2017). Application of marrow mesenchymal stem cell‐derived extracellular matrix in peripheral nerve tissue engineering. J. Tissue Eng. Regen. Med. 11 (8), 2250–2260. 10.1002/term.2123 26777754

[B23] Guillamat-Prats R. (2021). The role of MSC in wound healing, scarring and regeneration. Cells 10 (7), 1729. 10.3390/cells10071729 34359898 PMC8305394

[B24] Hassan N. H. Sulong A. F. Ng M. H. Htwe O. Idrus R. B. Roohi S. (2012). Neural‐differentiated mesenchymal stem cells incorporated into muscle stuffed vein scaffold forms a stable living nerve conduit. J. Orthop. Res. 30 (10), 1674–1681. 10.1002/jor.22102 22411691

[B25] Hopf A. Al-Bayati L. Schaefer D. J. Kalbermatten D. F. Guzman R. Madduri S. (2022). Optimized decellularization protocol for large peripheral nerve segments: towards personalized nerve bioengineering. Bioeng. (Basel) 9 (9), 412. 10.3390/bioengineering9090412 PMC9495622 36134958

[B26] Houshyar S. Bhattacharyya A. Shanks R. (2019). Peripheral nerve conduit: materials and structures. ACS Chem. Neurosci. 10 (8), 3349–3365. 10.1021/acschemneuro.9b00203 31273975

[B27] Hu S. L. Zhang J. Q. Hu X. Hu R. Luo H. S. Li F. (2009). *In vitro* labeling of human umbilical cord mesenchymal stem cells with superparamagnetic iron oxide nanoparticles. J. Cell. Biochem. 108 (2), 529–535. 10.1002/jcb.22283 19623584

[B28] Hu T. Chang S. Qi F. Zhang Z. Chen J. Jiang L. (2023). Neural grafts containing exosomes derived from Schwann cell-like cells promote peripheral nerve regeneration in rats. Burns Trauma 11, tkad013. 10.1093/burnst/tkad013 37122841 PMC10141455

[B29] Hu X. Wang X. Xu Y. Li L. Liu J. He Y. (2020). Electric conductivity on aligned nanofibers facilitates the transdifferentiation of mesenchymal stem cells into schwann cells and regeneration of injured peripheral nerve. Adv. Healthc. Mater. 9 (11), 1901570. 10.1002/adhm.201901570 32338461

[B30] Ikebe C. Suzuki K. (2014). Mesenchymal stem cells for regenerative therapy: optimization of cell preparation protocols. BioMed Res. Int. 2014 (1), 1–11. 10.1155/2014/951512 PMC3912818 24511552

[B31] Isern J. García-García A. Martín A. M. Arranz L. Martín-Pérez D. Torroja C. (2014). The neural crest is a source of mesenchymal stem cells with specialized hematopoietic stem cell niche function. Elife 3, e03696. 10.7554/elife.03696 25255216 PMC4381911

[B32] Jackson W. M. Nesti L. J. Tuan R. S. (2010). Potential therapeutic applications of muscle-derived mesenchymal stem and progenitor cells. Expert Opin. Biol. Ther. 10 (4), 505–517. 10.1517/14712591003610606 20218920 PMC3018682

[B33] Jankovic M. G. Stojkovic M. Bojic S. Jovicic N. Kovacevic M. M. Ivosevic Z. (2023). Scaling up human mesenchymal stem cell manufacturing using bioreactors for clinical uses. Curr. Res. Transl. Med. 71, 103393. 10.1016/j.retram.2023.103393 37163885

[B34] Jeon B.-G. Jang S.-J. Park J.-S. Subbarao R. B. Jeong G.-J. Park B.-W. (2015). Differentiation potential of mesenchymal stem cells isolated from human dental tissues into non-mesodermal lineage. Animal Cells Syst. 19 (5), 321–331. 10.1080/19768354.2015.1087430

[B35] Jiang H. Qian Y. Fan C. Ouyang Y. (2020). Polymeric guide conduits for peripheral nerve tissue engineering. Front. Bioeng. Biotechnol. 8, 582646. 10.3389/fbioe.2020.582646 33102465 PMC7546820

[B36] Kaizawa Y. Kakinoki R. Ikeguchi R. Ohta S. Noguchi T. Takeuchi H. (2017). A nerve conduit containing a vascular bundle and implanted with bone marrow stromal cells and decellularized allogenic nerve matrix. Cell Transplant. 26 (2), 215–228. 10.3727/096368916x692951 27657936 PMC5657762

[B37] Karimi S. Bagher Z. Najmoddin N. Simorgh S. Pezeshki-Modaress M. (2021). Alginate-magnetic short nanofibers 3D composite hydrogel enhances the encapsulated human olfactory mucosa stem cells bioactivity for potential nerve regeneration application. Int. J. Biol. Macromol. 167, 796–806. 10.1016/j.ijbiomac.2020.11.199 33278440

[B38] Kassis I. Vaknin-Dembinsky A. Karussis D. (2011). Bone marrow mesenchymal stem cells: agents of immunomodulation and neuroprotection. Curr. stem Cell Res. Ther. 6 (1), 63–68. 10.2174/157488811794480762 20955154

[B39] Kawashima N. Noda S. Yamamoto M. Okiji T. (2017). Properties of dental pulp–derived mesenchymal stem cells and the effects of culture conditions. J. Endod. 43 (9), S31–S34. 10.1016/j.joen.2017.06.004 28781092

[B40] Kim E. Y. Lee K.-B. Kim M. K. (2014). The potential of mesenchymal stem cells derived from amniotic membrane and amniotic fluid for neuronal regenerative therapy. BMB Rep. 47 (3), 135–140. 10.5483/bmbrep.2014.47.3.289 24499672 PMC4163884

[B41] Kim H. J. Lee J. S. Park J. M. Lee S. Hong S. J. Park J. S. (2020). Fabrication of nanocomposites complexed with gold nanoparticles on polyaniline and application to their nerve regeneration. ACS Appl. Mater. Interfaces 12 (27), 30750–30760. 10.1021/acsami.0c05286 32539331

[B42] Kornfeld T. Vogt P. M. Radtke C. (2019). Nerve grafting for peripheral nerve injuries with extended defect sizes. Wien. Med. Wochenschr. (1946) 169 (9), 240–251. 10.1007/s10354-018-0675-6 PMC6538587 30547373

[B43] Ladak A. Olson J. Tredget E. E. Gordon T. (2011). Differentiation of mesenchymal stem cells to support peripheral nerve regeneration in a rat model. Exp. Neurol. 228 (2), 242–252. 10.1016/j.expneurol.2011.01.013 21281630

[B44] Laroni A. de Rosbo N. K. Uccelli A. (2015). Mesenchymal stem cells for the treatment of neurological diseases: immunoregulation beyond neuroprotection. Immunol. Lett. 168 (2), 183–190. 10.1016/j.imlet.2015.08.007 26296458

[B45] Lavorato A. Raimondo S. Boido M. Muratori L. Durante G. Cofano F. (2021). Mesenchymal stem cell treatment perspectives in peripheral nerve regeneration: systematic review. Int. J. Mol. Sci. 22 (2), 572. 10.3390/ijms22020572 33430035 PMC7827385

[B46] Lee J. H. Lee J.-Y. Yang S. H. Lee E.-J. Kim H.-W. (2014). Carbon nanotube–collagen three-dimensional culture of mesenchymal stem cells promotes expression of neural phenotypes and secretion of neurotrophic factors. Acta Biomater. 10 (10), 4425–4436. 10.1016/j.actbio.2014.06.023 24954912

[B47] Li P. Gong Z. Shultz L. D. Ren G. (2019). Mesenchymal stem cells: from regeneration to cancer. Pharmacol. Ther. 200, 42–54. 10.1016/j.pharmthera.2019.04.005 30998940 PMC6626571

[B48] Li T. Xia M. Gao Y. Chen Y. Xu Y. (2015). Human umbilical cord mesenchymal stem cells: an overview of their potential in cell-based therapy. Expert Opin. Biol. Ther. 15 (9), 1293–1306. 10.1517/14712598.2015.1051528 26067213

[B49] Li X. Guan Y. Li C. Zhang T. Meng F. Zhang J. (2022). Immunomodulatory effects of mesenchymal stem cells in peripheral nerve injury. Stem Cell Res. Ther. 13 (1), 18–13. 10.1186/s13287-021-02690-2 35033187 PMC8760713

[B50] Liao I.-C. Wan H. Qi S. Cui C. Patel P. Sun W. (2013). Preclinical evaluations of acellular biological conduits for peripheral nerve regeneration. J. tissue Eng. 4, 204173141348103. 10.1177/2041731413481036 PMC3604911 23532671

[B51] Liu B. Kong Y. Shi W. Kuss M. Liao K. Hu G. (2022). Exosomes derived from differentiated human ADMSC with the Schwann cell phenotype modulate peripheral nerve-related cellular functions. Bioact. Mater. 14, 61–75. 10.1016/j.bioactmat.2021.11.022 35310346 PMC8892082

[B52] Liu P. Peng J. Han G.-H. Ding X. Wei S. Gao G. (2019). Role of macrophages in peripheral nerve injury and repair. Neural Regen. Res. 14 (8), 1335–1342. 10.4103/1673-5374.253510 30964051 PMC6524518

[B53] Llewellyn S. H. Faroni A. Iliut M. Bartlam C. Vijayaraghavan A. Reid A. J. (2021). Graphene oxide substrate promotes neurotrophic factor secretion and survival of human schwann‐like adipose mesenchymal stromal cells. Adv. Biol. 5 (4), 2000271. 10.1002/adbi.202000271 33852181

[B54] Lobov A. Kuchur P. Khizhina A. Kotova A. Ivashkin A. Kostina D. (2024). Mesenchymal cells retain the specificity of embryonal origin during osteogenic differentiation. Stem Cells 42 (1), 76–89. 10.1093/stmcls/sxad081 37931142

[B55] Lo Furno D. Mannino G. Giuffrida R. (2018). Functional role of mesenchymal stem cells in the treatment of chronic neurodegenerative diseases. J. Cell. physiology 233 (5), 3982–3999. 10.1002/jcp.26192 28926091

[B56] Ma Y. Dong L. Zhou D. Li L. Zhang W. Zhen Y. (2019). Extracellular vesicles from human umbilical cord mesenchymal stem cells improve nerve regeneration after sciatic nerve transection in rats. J. Cell. Mol. Med. 23 (4), 2822–2835. 10.1111/jcmm.14190 30772948 PMC6433678

[B57] Mannelli L. D. C. Tenci B. Micheli L. Vona A. Corti F. Zanardelli M. (2018). Adipose-derived stem cells decrease pain in a rat model of oxaliplatin-induced neuropathy: role of VEGF-A modulation. Neuropharmacology 131, 166–175. 10.1016/j.neuropharm.2017.12.020 29241656

[B58] Mao Q. Nguyen P. D. Shanti R. M. Shi S. Shakoori P. Zhang Q. (2019). Gingiva-derived mesenchymal stem cell-extracellular vesicles activate schwann cell repair phenotype and promote nerve regeneration. Tissue Eng. Part A 25 (11-12), 887–900. 10.1089/ten.tea.2018.0176 30311853

[B59] Marconi S. Castiglione G. Turano E. Bissolotti G. Angiari S. Farinazzo A. (2012). Human adipose-derived mesenchymal stem cells systemically injected promote peripheral nerve regeneration in the mouse model of sciatic crush. Tissue Eng. Part A 18 (11-12), 1264–1272. 10.1089/ten.tea.2011.0491 22332955

[B60] Martini R. Willison H. (2016). Neuroinflammation in the peripheral nerve: cause, modulator, or bystander in peripheral neuropathies? Glia 64 (4), 475–486. 10.1002/glia.22899 26250643 PMC4832258

[B61] Mathot F. Rbia N. Bishop A. T. Hovius S. E. R. Shin A. Y. (2020a). Adipose derived mesenchymal stem cells seeded onto a decellularized nerve allograft enhances angiogenesis in a rat sciatic nerve defect model. Microsurgery 40 (5), 585–592. 10.1002/micr.30579 32233045 PMC7570204

[B62] Mathot F. Rbia N. Thaler R. Bishop A. T. Van Wijnen A. J. Shin A. Y. (2020b). Gene expression profiles of differentiated and undifferentiated adipose derived mesenchymal stem cells dynamically seeded onto a processed nerve allograft. Gene 724, 144151. 10.1016/j.gene.2019.144151 31626959

[B63] Minteer D. Marra K. G. Rubin J. P. (2013). “Adipose-derived mesenchymal stem cells: biology and potential applications,” in Mesenchymal stem cells-basics and clinical application I. Editors Weyand B. Kasper C. Dominici M. Hass R. Jacobs R. (Springer Berlin Heidelberg), 59–71. 10.1007/10_2012_146 22825719

[B64] Moattari M. Moattari F. Kaka G. Mohseni Kouchesfehani H. Sadraie S. H. Naghdi M. (2018). Evaluation of dexamethasone treated mesenchymal stem cells for recovery in neurotmesis model of peripheral nerve injury. Neurological Res. 40 (12), 1060–1070. 10.1080/01616412.2018.1517859 30246623

[B65] Morikawa S. Mabuchi Y. Niibe K. Suzuki S. Nagoshi N. Sunabori T. (2009). Development of mesenchymal stem cells partially originate from the neural crest. Biochem. Biophysical Res. Commun. 379 (4), 1114–1119. 10.1016/j.bbrc.2009.01.031 19161980

[B66] Musavi L. Brandacher G. Hoke A. Darrach H. Lee W. A. Kumar A. (2018). Muscle-derived stem cells: important players in peripheral nerve repair. Expert Opin. Ther. targets 22 (12), 1009–1016. 10.1080/14728222.2018.1539706 30347175

[B67] Oliveira J. T. Bittencourt-Navarrete R. E. de Almeida F. M. Tonda-Turo C. Martinez A. M. B. Franca J. G. (2014). Enhancement of median nerve regeneration by mesenchymal stem cells engraftment in an absorbable conduit: improvement of peripheral nerve morphology with enlargement of somatosensory cortical representation. Front. Neuroanat. 8, 111. 10.3389/fnana.2014.00111 25360086 PMC4199278

[B68] Orciani M. Di Primio R. (2013). “Skin-derived mesenchymal stem cells: isolation, culture, and characterization,” in Skin stem cells: methods and protocols. Editor Turksen K. (Totowa, NJ: Humana Press), 275–283. 10.1007/978-1-62703-330-5_21 23483402

[B69] Ortiz A. d.C. Fideles S. O. M. Pomini K. T. Bellini M. Z. Pereira E. D. S. B. M. Reis C. H. B. (2022). Potential of fibrin glue and mesenchymal stem cells (MSCs) to regenerate nerve injuries: a systematic review. Cells 11 (2), 221. 10.3390/cells11020221 35053336 PMC8773549

[B70] Panagopoulos G. N. Megaloikonomos P. D. Mavrogenis A. F. (2017). The present and future for peripheral nerve regeneration. Orthopedics 40 (1), e141–e156. 10.3928/01477447-20161019-01 27783836

[B71] Park B. W. Kang D. H. Kang E. J. Byun J. H. Lee J. S. Maeng G. H. (2012). Peripheral nerve regeneration using autologous porcine skin‐derived mesenchymal stem cells. J. tissue Eng. Regen. Med. 6 (2), 113–124. 10.1002/term.404 21337707

[B72] Pinho A. C. Fonseca A. C. Serra A. C. Santos J. D. Coelho J. F. (2016). Peripheral nerve regeneration: current status and new strategies using polymeric materials. Adv. Healthc. Mater. 5 (21), 2732–2744. 10.1002/adhm.201600236 27600578

[B73] Pisciotta A. Bertoni L. Vallarola A. Bertani G. Mecugni D. Carnevale G. (2020). Neural crest derived stem cells from dental pulp and tooth-associated stem cells for peripheral nerve regeneration. Neural Regen. Res. 15 (3), 373. 10.4103/1673-5374.266043 31571644 PMC6921350

[B74] Qian Y. Song J. Zheng W. Zhao X. Ouyang Y. Yuan W. (2018). 3D manufacture of gold nanocomposite channels facilitates neural differentiation and regeneration. Adv. Funct. Mater. 28 (14), 1707077. 10.1002/adfm.201707077

[B75] Ramli K. Gasim A. I. Ahmad A. A. Htwe O. Mohamed Haflah N. H. Law Z. K. (2019). Efficacy of human cell-seeded muscle-stuffed vein conduit in rat sciatic nerve repair. Tissue Eng. Part A 25 (19-20), 1438–1455. 10.1089/ten.tea.2018.0279 30848172

[B76] Rao F. Zhang D. Fang T. Lu C. Wang B. Ding X. (2019). Exosomes from human gingiva-derived mesenchymal stem cells combined with biodegradable chitin conduits promote rat sciatic nerve regeneration. Stem cells Int. 2019, 1–12. 10.1155/2019/2546367 PMC6525800 31191669

[B77] Rbia N. Bulstra L. F. Lewallen E. A. Hovius S. E. van Wijnen A. J. Shin A. Y. (2019a). Seeding decellularized nerve allografts with adipose-derived mesenchymal stromal cells: an *in vitro* analysis of the gene expression and growth factors produced. J. Plastic, Reconstr. Aesthetic Surg. 72 (8), 1316–1325. 10.1016/j.bjps.2019.04.014 31175032

[B78] Rbia N. Bulstra L. F. Thaler R. Hovius S. E. van Wijnen A. J. Shin A. Y. (2019b). *In vivo* survival of mesenchymal stromal cell–enhanced decellularized nerve grafts for segmental peripheral nerve reconstruction. J. hand Surg. 44 (6), 514.e1–514.e11. 10.1016/j.jhsa.2018.07.010 30301645

[B79] Ribeiro J. Pereira T. Caseiro A. R. Armada-da-Silva P. Pires I. Prada J. (2015). Evaluation of biodegradable electric conductive tube-guides and mesenchymal stem cells. World J. Stem Cells 7 (6), 956–975. 10.4252/wjsc.v7.i6.956 26240682 PMC4515438

[B80] Ribitsch I. Baptista P. M. Lange-Consiglio A. Melotti L. Patruno M. Jenner F. (2020). Large animal models in regenerative medicine and tissue engineering: to do or not to do. Front. Bioeng. Biotechnol. 8, 972. 10.3389/fbioe.2020.00972 32903631 PMC7438731

[B81] Rodríguez-Sánchez D. N. Pinto G. B. A. Cartarozzi L. P. de Oliveira A. L. R. Bovolato A. L. C. de Carvalho M. (2021). 3D-printed nerve guidance conduits multi-functionalized with canine multipotent mesenchymal stromal cells promote neuroregeneration after sciatic nerve injury in rats. Stem Cell Res. Ther. 12 (1), 303. 10.1186/s13287-021-02315-8 34051869 PMC8164252

[B82] Saffari S. Saffari T. M. Chan K. Borschel G. H. Shin A. Y. (2021a). Mesenchymal stem cells and local tacrolimus delivery synergistically enhance neurite extension. Biotechnol. Bioeng. 118 (11), 4477–4487. 10.1002/bit.27916 34396506 PMC8744499

[B83] Saffari T. M. Chan K. Saffari S. Zuo K. J. McGovern R. M. Reid J. M. (2021b). Combined local delivery of tacrolimus and stem cells in hydrogel for enhancing peripheral nerve regeneration. Biotechnol. Bioeng. 118 (7), 2804–2814. 10.1002/bit.27799 33913523

[B84] Sanchez D. N. R. Bertanha M. Fernandes T. D. de Lima Resende L. A. Deffune E. Amorim R. M. (2017). Effects of canine and murine mesenchymal stromal cell transplantation on peripheral nerve regeneration. Int. J. Stem Cells 10 (1), 83–92. 10.15283/ijsc16037 28446003 PMC5488780

[B85] Scheib J. Höke A. (2013). Advances in peripheral nerve regeneration. Nat. Rev. Neurol. 9 (12), 668–676. 10.1038/nrneurol.2013.227 24217518

[B86] Sensharma P. Madhumathi G. Jayant R. D. Jaiswal A. K. (2017). Biomaterials and cells for neural tissue engineering: current choices. Mater. Sci. Eng. C 77, 1302–1315. 10.1016/j.msec.2017.03.264 28532008

[B87] Sezer G. Yay A. H. Sarica Z. S. Gonen Z. B. Onder G. O. Alan A. (2022). Bone marrow-derived mesenchymal stem cells alleviate paclitaxel-induced mechanical allodynia in rats. J. Biochem. Mol. Toxicol. 36 (12), e23207. 10.1002/jbt.23207 36052563

[B88] Shalaby S. M. Amal S. Ahmed F. E. Shaban S. F. Wahdan R. A. Kandel W. A. (2017). Combined Wharton’s jelly derived mesenchymal stem cells and nerve guidance conduit: a potential promising therapy for peripheral nerve injuries. Int. J. Biochem. Cell Biol. 86, 67–76. 10.1016/j.biocel.2017.03.002 28274689

[B89] Sharifi M. Cho W. C. Ansariesfahani A. Tarharoudi R. Malekisarvar H. Sari S. (2022a). An updated review on EPR-based solid tumor targeting nanocarriers for cancer treatment. Cancers 14 (12), 2868. 10.3390/cancers14122868 35740534 PMC9220781

[B90] Sharifi M. Farahani M. K. Salehi M. Atashi A. Alizadeh M. Kheradmandi R. (2022b). Exploring the physicochemical, electroactive, and biodelivery properties of metal nanoparticles on peripheral nerve regeneration. ACS Biomaterials Sci. Eng. 9 (1), 106–138. 10.1021/acsbiomaterials.2c01216 36545927

[B91] Sharifi M. Kamalabadi-Farahani M. Salehi M. Ebrahimi-Barough S. Alizadeh M. (2024). Recent advances in enhances peripheral nerve orientation: the synergy of micro or nano patterns with therapeutic tactics. J. Nanobiotechnology 22 (1), 194. 10.1186/s12951-024-02475-8 38643117 PMC11031871

[B92] Sharifi M. Kheradmandi R. Salehi M. Alizadeh M. ten Hagen T. L. M. Falahati M. (2022c). Criteria, challenges, and opportunities for acellularized allogeneic/xenogeneic bone grafts in bone repairing. ACS Biomaterials Sci. Eng. 8 (8), 3199–3219. 10.1021/acsbiomaterials.2c00194 35816626

[B93] Singh A. Raghav A. Shiekh P. A. Kumar A. (2021). Transplantation of engineered exosomes derived from bone marrow mesenchymal stromal cells ameliorate diabetic peripheral neuropathy under electrical stimulation. Bioact. Mater. 6 (8), 2231–2249. 10.1016/j.bioactmat.2021.01.008 33553812 PMC7829156

[B94] Soto P. A. Vence M. Piñero G. M. Coral D. F. Usach V. Muraca D. (2021). Sciatic nerve regeneration after traumatic injury using magnetic targeted adipose-derived mesenchymal stem cells. Acta Biomater. 130, 234–247. 10.1016/j.actbio.2021.05.050 34082099

[B95] Sramkó B. Földes A. Kádár K. Varga G. Zsembery Á. Pircs K. (2023). The wisdom in teeth: neuronal differentiation of dental pulp cells. Cell. Reprogr. 25 (1), 32–44. 10.1089/cell.2022.0102 PMC9963504 36719998

[B96] Srinivasan A. Chang S.-Y. Zhang S. Toh W. S. Toh Y.-C. (2018). Substrate stiffness modulates the multipotency of human neural crest derived ectomesenchymal stem cells via CD44 mediated PDGFR signaling. Biomaterials 167, 153–167. 10.1016/j.biomaterials.2018.03.022 29571051

[B97] Sullivan R. Dailey T. Duncan K. Abel N. Borlongan C. V. (2016). Peripheral nerve injury: stem cell therapy and peripheral nerve transfer. Int. J. Mol. Sci. 17 (12), 2101. 10.3390/ijms17122101 27983642 PMC5187901

[B98] Sun A. X. Prest T. A. Fowler J. R. Brick R. M. Gloss K. M. Li X. (2019). Conduits harnessing spatially controlled cell-secreted neurotrophic factors improve peripheral nerve regeneration. Biomaterials 203, 86–95. 10.1016/j.biomaterials.2019.01.038 30857644

[B99] Sun F. Zhou K. Mi W.-j. Qiu J.-h. (2011a). Combined use of decellularized allogeneic artery conduits with autologous transdifferentiated adipose-derived stem cells for facial nerve regeneration in rats. Biomaterials 32 (32), 8118–8128. 10.1016/j.biomaterials.2011.07.031 21816463

[B100] Sun F. Zhou K. Mi W.-j. Qiu J.-h. (2011b). Repair of facial nerve defects with decellularized artery allografts containing autologous adipose-derived stem cells in a rat model. Neurosci. Lett. 499 (2), 104–108. 10.1016/j.neulet.2011.05.043 21651959

[B101] Supra R. Wilson D. R. Agrawal D. K. (2023). Therapeutic potential of “smart” exosomes in peripheral nerve regeneration. J. Biotechnol. Biomed. 6, 189–196. 10.26502/jbb.2642-91280082 37388677 PMC10310314

[B102] Todd T. (1823). On the process of reproduction of the members of the aquatic salamander. Quart. J. Sci. Arts Lib. 16, 84–86.

[B103] Tseng T.-C. Hsu S.-h. (2014). Substrate-mediated nanoparticle/gene delivery to MSC spheroids and their applications in peripheral nerve regeneration. Biomaterials 35 (9), 2630–2641. 10.1016/j.biomaterials.2013.12.021 24388817

[B104] Uz M. Donta M. Mededovic M. Sakaguchi D. S. Mallapragada S. K. (2019). Development of gelatin and graphene-based nerve regeneration conduits using three-dimensional (3D) printing strategies for electrical transdifferentiation of mesenchymal stem cells. Industrial Eng. Chem. Res. 58 (18), 7421–7427. 10.1021/acs.iecr.8b05537

[B105] Uz M. Hondred J. A. Donta M. Jung J. Kozik E. Green J. (2020). Determination of electrical stimuli parameters to transdifferentiate genetically engineered mesenchymal stem cells into neuronal or glial lineages. Regen. Eng. Transl. Med. 6, 18–28. 10.1007/s40883-019-00126-1

[B106] Uzun T. Toptas O. Saylan A. Carver H. Turkoglu S. A. (2019). Evaluation and comparison of the effects of artesunate, dexamethasone, and tacrolimus on sciatic nerve regeneration. J. Oral Maxillofac. Surg. 77 (5), 1092.e1–1092.e12. 10.1016/j.joms.2018.12.019 30689960

[B107] Vijayavenkataraman S. (2020). Nerve guide conduits for peripheral nerve injury repair: a review on design, materials and fabrication methods. Acta Biomater. 106, 54–69. 10.1016/j.actbio.2020.02.003 32044456

[B108] Volkman R. Offen D. (2017). Concise review: mesenchymal stem cells in neurodegenerative diseases. Stem cells 35 (8), 1867–1880. 10.1002/stem.2651 28589621

[B109] Wang H. Jia Y. Li J. Liu Q. (2020). Schwann cell-derived exosomes induce bone marrow-derived mesenchymal stem cells to express Schwann cell markers *in vitro* . Mol. Med. Rep. 21 (3), 1640–1646. 10.3892/mmr.2020.10960 32016464

[B110] Wang X. H. Guo J. S. Chen S. L. Dai H. L. Ding J. X. Han N. (2015). Repair, protection and regeneration of peripheral nerve injury. Neural Regen. Res. 10 (11), 1777–1798. 10.4103/1673-5374.170301 26807113 PMC4705790

[B111] Wang Y. H. Guo Y. C. Wang D. R. Liu J. Y. Pan J. (2019). Adipose stem cell-based clinical strategy for neural regeneration: a review of current opinion. Stem Cells Int. 2019, 1–10. 10.1155/2019/8502370 PMC6885831 31827536

[B112] Wang Y. Zhao Z. Ren Z. Zhao B. Zhang L. Chen J. (2012). Recellularized nerve allografts with differentiated mesenchymal stem cells promote peripheral nerve regeneration. Neurosci. Lett. 514 (1), 96–101. 10.1016/j.neulet.2012.02.066 22405891

[B113] Widgerow A. D. Salibian A. A. Lalezari S. Evans G. R. (2013). Neuromodulatory nerve regeneration: adipose tissue‐derived stem cells and neurotrophic mediation in peripheral nerve regeneration. J. Neurosci. Res. 91 (12), 1517–1524. 10.1002/jnr.23284 24105674 PMC7061900

[B114] Wu S. Qi Y. Shi W. Kuss M. Chen S. Duan B. (2022). Electrospun conductive nanofiber yarns for accelerating mesenchymal stem cells differentiation and maturation into Schwann cell-like cells under a combination of electrical stimulation and chemical induction. Acta Biomater. 139, 91–104. 10.1016/j.actbio.2020.11.042 33271357 PMC8164650

[B115] Xu X. Chen C. Akiyama K. Chai Y. Le A. Wang Z. (2013). Gingivae contain neural-crest-and mesoderm-derived mesenchymal stem cells. J. Dent. Res. 92 (9), 825–832. 10.1177/0022034513497961 23867762 PMC3744273

[B116] Yao X. Yan Z. Li X. Li Y. Ouyang Y. Fan C. (2021). Tacrolimus-induced neurotrophic differentiation of adipose-derived stem cells as novel therapeutic method for peripheral nerve injury. Front. Cell. Neurosci. 15, 799151. 10.3389/fncel.2021.799151 34955758 PMC8692949

[B117] Yu T. Xu Y. Ahmad M. A. Javed R. Hagiwara H. Tian X. (2021). Exosomes as a promising therapeutic strategy for peripheral nerve injury. Curr. Neuropharmacol. 19 (12), 2141–2151. 10.2174/1570159x19666210203161559 33535957 PMC9185764

[B118] Zack-Williams S. D. Butler P. E. Kalaskar D. M. (2015). Current progress in use of adipose derived stem cells in peripheral nerve regeneration. World J. stem cells 7 (1), 51. 10.4252/wjsc.v7.i1.51 25621105 PMC4300936

[B119] Zhang H. Guo J. Wang Y. Shang L. Chai R. Zhao Y. (2022). Natural polymer‐derived bioscaffolds for peripheral nerve regeneration. Adv. Funct. Mater. 32 (41), 2203829. 10.1002/adfm.202203829

[B120] Zhang Q. Nguyen P. Burrell J. C. Zeng J. Shi S. Shanti R. M. (2021a). Harnessing 3D collagen hydrogel-directed conversion of human GMSCs into SCP-like cells to generate functionalized nerve conduits. NPJ Regen. Med. 6 (1), 59. 10.1038/s41536-021-00170-y 34593823 PMC8484485

[B121] Zhang R.-C. Du W.-Q. Zhang J.-Y. Yu S.-X. Lu F.-Z. Ding H.-M. (2021b). Mesenchymal stem cell treatment for peripheral nerve injury: a narrative review. Neural Regen. Res. 16 (11), 2170. 10.4103/1673-5374.310941 33818489 PMC8354135

[B122] Zhang W. Fang X. X. Li Q. C. Pi W. Han N. (2023). Reduced graphene oxide-embedded nerve conduits loaded with bone marrow mesenchymal stem cell-derived extracellular vesicles promote peripheral nerve regeneration. Neural Regen. Res. 18 (1), 200–206. 10.4103/1673-5374.343889 35799543 PMC9241414

[B123] Zhang X. Qu W. Li D. Shi K. Li R. Han Y. (2020a). Functional polymer-based nerve guide conduits to promote peripheral nerve regeneration. Adv. Mater. Interfaces 7 (14), 2000225. 10.1002/admi.202000225

[B124] Zhang Y. Wang W.-T. Gong C.-R. Li C. Shi M. (2020b). Combination of olfactory ensheathing cells and human umbilical cord mesenchymal stem cell-derived exosomes promotes sciatic nerve regeneration. Neural Regen. Res. 15 (10), 1903. 10.4103/1673-5374.280330 32246639 PMC7513967

[B125] Zhao Z. Wang Y. Peng J. Ren Z. Zhan S. Liu Y. (2011). Repair of nerve defect with acellular nerve graft supplemented by bone marrow stromal cells in mice. Microsurgery 31 (5), 388–394. 10.1002/micr.20882 21503972

[B126] Zheng L. Cui H.-F. (2012). Enhancement of nerve regeneration along a chitosan conduit combined with bone marrow mesenchymal stem cells. J. Mater. Sci. Mater. Med. 23, 2291–2302. 10.1007/s10856-012-4694-3 22661248

[B127] Zheng Y. Hasan A. Babadaei M. M. N. Behzadi E. Nouri M. Sharifi M. (2020). Exosomes: multiple-targeted multifunctional biological nanoparticles in the diagnosis, drug delivery, and imaging of cancer cells. Biomed. Pharmacother. 129, 110442. 10.1016/j.biopha.2020.110442 32593129

[B128] Zheng Y. Huang C. Liu F. Lin H. Niu Y. Yang X. (2018). Reactivation of denervated Schwann cells by neurons induced from bone marrow-derived mesenchymal stem cells. Brain Res. Bull. 139, 211–223. 10.1016/j.brainresbull.2018.03.005 29524470

[B129] Zhou N. Xu Z. Li X. Ren S. Chen J. Xiong H. (2022). Schwann cell-derived exosomes induce the differentiation of human adipose-derived stem cells into schwann cells. Front. Mol. Biosci. 8, 835135. 10.3389/fmolb.2021.835135 35174212 PMC8841477

[B130] Zhu L. Liu T. Cai J. Ma J. Chen A.-m. (2015). Repair and regeneration of lumbosacral nerve defects in rats with chitosan conduits containing bone marrow mesenchymal stem cells. Injury 46 (11), 2156–2163. 10.1016/j.injury.2015.08.035 26429103

[B131] Zuo K. J. Gordon T. Chan K. M. Borschel G. H. (2020). Electrical stimulation to enhance peripheral nerve regeneration: update in molecular investigations and clinical translation. Exp. Neurol. 332, 113397. 10.1016/j.expneurol.2020.113397 32628968

